# Explainable Intrusion and Anomaly Detection for IoT Sensor Networks Using Hybrid Feature Selection and Deep Autoencoder Learning

**DOI:** 10.3390/s26144540

**Published:** 2026-07-17

**Authors:** Usman Ahmed, Sadiq Muhammad, Jaeyoung Choi

**Affiliations:** 1 School of Software, Northwestern Polytechnical University, Changan Campus, Xi’an 710072, China; 2 School of Computing, Gachon University, Seongnam-si 13120, Republic of Korea

**Keywords:** deep autoencoders, explainable AI, feedforward neural networks, feature selection, intrusion detection

## Abstract

Internet of Things (IoT) environments, which are distributed and resource-constrained, present unique security challenges, making it essential to develop robust and transparent intrusion detection system (IDS) solutions. This study presents a specialized system for IoT environments that combines hybrid feature selection methods with anomaly detection algorithms and classification strategies, alongside explainability techniques, to enhance security measures and event transparency. The novelty of this work lies in combining several modern approaches: hybrid feature selection by combining Random Forest (RF) and SelectKBest to reduce computational overhead while preserving high accuracy of detection; the application of Deep Autoencoders (DAEs) for detecting anomalous traffic deviating from learned normal behavior, enabling detection of previously unseen attack patterns under controlled experimental conditions; feedforward neural networks (FNNs) are applied to classify anomalous data with high accuracy and reduced training time, and explainability tools such as Shapley Additive Explanations (SHAP) and Local Interpretable Model-Agnostic Explanations (LIME) are incorporated to provide insights into model decisions and improve trust. We utilize the CIC-IDS2017 and EIIoT datasets to evaluate their effectiveness in identifying critical cyber threats and subsequently classifying them. This proposed framework, HDATL-XAI (Hybrid Dimension-reduction Autoencoder and Traditional Learning with Explainable Artificial Intelligence), integrates advanced techniques to offer comprehensive protection while ensuring transparency, enhancing security and trustworthiness, and serving as an essential tool for protecting IoT networks.

## 1. Introduction

The rise of Internet of Things (IoT) devices [1] has transformed everyday routines across numerous sectors, including smart homes and industrial automation. By 2025, the number of connected IoT devices is projected to exceed 75 billion globally, expanding the attack surface available to adversaries at an unprecedented scale [2]. Ref. [2] provides a comprehensive taxonomy of IoT security threats, identifying resource constraints, heterogeneous communication protocols, and the absence of standardized security frameworks as the primary factors that make IoT networks structurally more vulnerable than conventional IT infrastructure. As a large number of IoT devices rapidly connect, significant vulnerabilities in their security systems have emerged, making IoT networks increasingly common targets of cybercriminals [3]. IDSs and IPSs have become essential tools for protecting various network environments. IDS monitors intrusion activities, while IPS takes proactive steps to prevent intrusion when they are identified [4].

The risk of cyberattacks against Internet of Things (IoT) devices continues to grow as they proliferate across sectors such as health care, smart cities, and industrial automation. This heightened risk stems largely from the sheer number and decentralized distribution of potential attack points, which give attackers numerous entry paths into a network [5]. Traditional security solutions have been largely unsuccessful in protecting IoT systems because they typically demand significant processing power, rely on specific network communication protocols, or both; consequently, no general-purpose IDS is currently available that can be effectively deployed across IoT environments. This motivates the development of IDSs that provide strong protection while operating within the minimal resource budgets typical of IoT systems [6].

These complex, heterogeneous IoT environments pose new challenges for intrusion detection and prevention. Traditional network-based IDSs often fail to detect complex attacks, such as file-less malware, which require more advanced solutions developed with a specific focus [7]. Recently, advances in Artificial Intelligence (AI), particularly machine learning (ML) and deep learning (DL), have significantly enhanced the capabilities of IDS/IPS systems. By analyzing large volumes of traffic data to identify patterns indicative of malicious behavior, these technologies have improved both detection accuracy and response times [8].

Another concern in IoT security is the rise in zero-day attacks (ZDAs), previously unknown vulnerabilities that attackers can exploit before a patch is released to address them. Signature-based detection methods typically rely on predefined attack signatures and thus cannot detect new types of threats. Given the continuously escalating landscape of cyberattacks, IoT networks require an IDS capable of detecting abnormal behavior, even if it does not match known attack patterns. This demonstrates the need for more advanced machine learning-driven techniques to identify both known and unknown threats through behavioral analysis [9,10].

This study addresses these challenges by proposing a secure ensemble-based intrusion-detection and network-traffic inspection framework for IoT environments. The system proposed herein uses Random Forest (RF) and SelectKBest for hybrid feature selection, ensuring that the most relevant features are retained from a large dataset of IoT traffic. By selecting only the critical features, this hybrid approach reduces computational load and memory usage while simultaneously enhancing anomaly detection accuracy.

Attacks in IoT contexts have varied, ranging from denial-of-service (DoS/DDoS) and distributed denial-of-service (DDoS) attacks to man-in-the-middle (MITM) and malware attacks [11,12]. This complexity requires the IDS to detect threats and correctly classify them to trigger appropriate responses. Feedforward neural networks (FNNs) are incorporated into our solution to make the system more adaptable. Another important obstacle in developing IDS solutions for IoT is the reliance on uninterpretable models built from a variety of machine learning techniques. The proposed system has included SHAP and LIME to give an explanation for the decisions made by the IDS system. Both of these interpretive tools show what factors are most important (feature selection), provide a local interpretation for each prediction made by the IDS system, and provide great insight to help increase transparency and confidence in the IDS system.

### 1.1. Research Contributions

**Figure 1:**
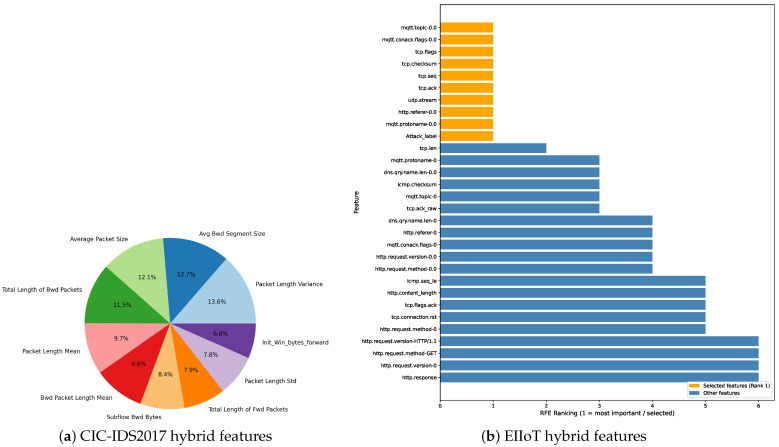
Hybrid feature selection using RF and SelectKBest. Selected features are listed in order of importance score; label overlap in the original visualization has been resolved by restricting display to the top-15 features per method for readability.

**Figure 2:**
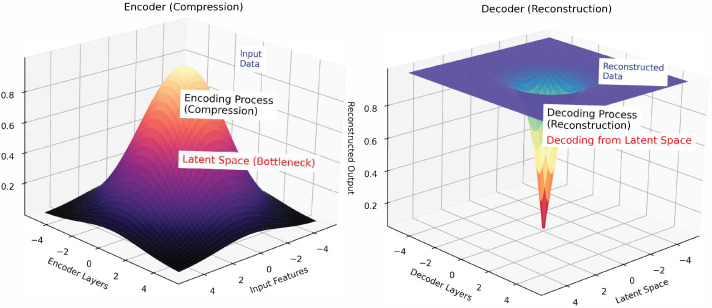
Overview of the deep autoencoder encoder–decoder structure.

**Figure 3:**
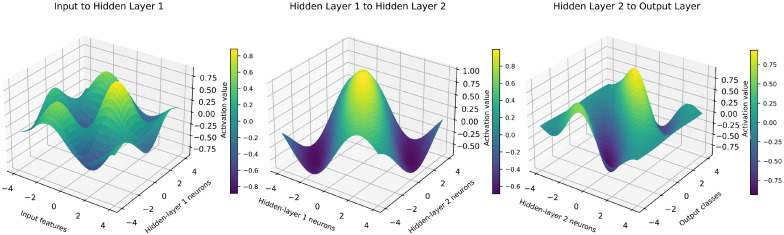
FNN functional transformations across hidden layers. Colorbars indicate activation magnitude.

Previous IDS techniques have typically optimized for either detection effectiveness or processing speed, rarely both. This research improves upon such approaches by combining a hybrid detection technique with explainability, achieving both effectiveness and speed while also revealing how the IDS arrives at each decision. This added interpretability is valuable in real-world cybersecurity applications, where understanding why an event was flagged as an attack is often as important as the detection itself:

- The (IDS) employs a hybrid feature selection methodology (Random Forest and SelectKBest) on CIC-IDS2017 and EIIoT datasets to identify critical features, enhancing efficiency in resource-constrained IoT environments, as shown in Figure 1.
- The DAE in Figure 2 learns the distribution of normal traffic and flags deviations as anomalies; this enables detection of attack patterns absent from the training label set, approximating zero-day detection under standard train/test evaluation conditions.
- Detected anomalies are classified using an FNN as shown in Figure 3 for accurate threat categorization and prompt response.
- Explainability techniques like SHAP and LIME are incorporated to improve trustworthiness and transparency by clarifying feature importance and individual prediction explanations.

While each constituent technique is individually established, the technical novelty of HDATL-XAI lies in three specific design decisions that distinguish it from prior integration attempts. First, the hybrid feature selector (RF + SelectKBest) combines a nonlinear importance ranker with a univariate filter, explicitly resolving the tension between multicollinearity suppression and statistical relevance, a combination not evaluated on these datasets in prior XAI-IDS work. Second, the DAE is trained exclusively on normal-class samples from the post-SMOTE training partition, ensuring the reconstruction threshold $\tau^{*}$ is optimized on a held-out validation subset without test-set leakage, a stricter protocol than most autoencoder-based IDS systems. Third, rather than applying SHAP/LIME as a post hoc visualization afterthought, this framework uses SHAP feature rankings to cross-validate the output of the hybrid selector, providing a closed-loop verification that selected features are both statistically relevant and security-meaningful. Together, these decisions constitute a co-designed pipeline rather than a loose assembly of existing tools.

### 1.2. Paper Organization

The rest of the paper is organized as follows: Section 2 reviews related work. Section 3 discusses the datasets and selection process. Section 4 details the preprocessing and proposed method, including the complete architecture, method formulation, FNN transformation, and experimental setup. Section 5 covers the confusion matrix, validation analysis, statistical tests, Explainable AI analysis using SHAP and LIME, and discussion, including performance analysis and comparisons with state-of-the-art methods. Finally, Section 6 concludes the work and addresses data availability.

## 2. Related Work

Traditional IDS models use a signature-based method to compare each incoming packet with known “signatures” or “attack patterns”. The limitation of this approach is that it cannot identify new forms of malware or previously unseen threats. Consequently, researchers have increasingly turned to developing more adaptable IDS systems based on machine learning models [13,14,15]. In addition, while newer IDS models (such as those using Deep Autoencoders (DAEs) and transfer learning) [16] improve detection accuracy, they can also pose challenges for security professionals who need to understand how the system made its decisions. Techniques like SHAP and LIME provide explanations of the features of the input data that the system uses to make its decision [17]. Another area of research focuses on developing lightweight solutions for processing large volumes of IoT sensor data streams to achieve real-time intrusion detection without imposing excessive computational burden [18].

Recent advances in anomaly-based intrusion detection systems (IDSs) have led to the development of systems capable of detecting unknown or “zero-day” attacks by identifying deviations from normal network traffic behavior [19]. Researchers have utilized Deep Autoencoders for anomaly detection because they can model a wide range of normal traffic behavior through pattern reconstruction [20]. The resulting error in reconstructing traffic can be used to identify anomalies. Deep learning is capable of detecting unknown or “zero-day” attacks by identifying deviations in encoders’ use of unsupervised learning, allowing them to learn patterns of benign traffic activity without access to labeled attack data. Because new attack methods emerge rapidly in IoT devices, this method is especially useful for IoT applications. Various studies have shown that Deep Autoencoder methods generalize very well across different network types and environments when applied to zero-day attack detection [21]. In addition, recent research has demonstrated that combining AI and blockchain technology (such as Ethereum and IPFS) can provide a strong foundation for secure, tamper-proof anomaly detection in IoT and healthcare applications [22]. These techniques address many of the data privacy concerns associated with trusted decision-making in high-risk areas. As in other applications, researchers have implemented AI-driven intrusion detection systems (IDSs), specifically those using convolutional neural networks (CNNs) and support vector machines (SVMs) for anomaly detection in the Health Care IoT (IoHT). Their results highlight an increasing reliance on AI to protect IoHT infrastructures [23].

The information in Table 1 provides a general overview of examples of Internet-of-Things (IoT)-based intrusion detection systems published between 2022 and 2025. In addition to the applied dataset and method, this table also includes the achieved accuracy and the IDS classification type and category. The datasets were selected from several common benchmark datasets, such as UNSW-NB15, NSL-KDD, ToN_IoT, and CIC-IDS2017, as well as IoT-specific datasets, such as CICIoT2023, IoTID20, and BoT-IoT. Traditional machine learning algorithms such as support vector machines, Random Forest, and feature selection-driven models are among the most commonly used. In addition, hybrid models combining k-nearest neighbors, decision trees, and logistic regression have become very popular. Even newer architectures, like federated learning with long short-term memory networks, transformer models combined with convolutional neural networks, bidirectional long short-term memory networks, and explainable deep learning models using Shapley values or local interpretable model explanations, have gained increasing popularity.

Explainability has emerged as a dominant trend in recent works. Among the 2024–2025 entries, Alabbadi and Bajaber [24] proposed a 1D-CNN and DNN architecture evaluated on TON_IoT with SHAP and LIME, achieving 99.24% accuracy. Gaspar et al. [25] applied LIME and SHAP to a Multi-Layer Perceptron for IoT device intrusion detection, reporting 93.71% accuracy in a binary classification setting. Anwar et al. [26] demonstrated the applicability of Federated Learning combined with LSTM on the CIC-IDS2017 and UNSW-NB15 datasets, achieving 98.10% multi-class accuracy while preserving data privacy. Zhang et al. [27] introduced a hybrid Transformer and CNN-BiLSTM architecture evaluated on CIC-IDS2017 and BoT-IoT, reaching 98.76% accuracy with strong generalization across attack categories.

**Table 1:** Summary of Related Work on intrusion detection systems for IoT.

Reference	Year	Dataset	Method	Accuracy	Classification	Type
[28]	2022	UNSW-NB15	Snort IDS	N/A	Binary	SDN-IoT
[29]	2022	IoTID20, NSL-KDD	Feature selection (IG/GR)	99.98%	Binary	ML-based IDS
[30]	2022	NSL-KDD	Random Forest	99.3%	Binary/Multi-class	Ensemble learning
[31]	2022	ToN_IoT	ReliefF-based ML	98.39%	Multi-class	Deep learning
[32]	2023	CICIoT2023, IoTID20	Blending model + LIME	N/A	Multi-class	XAI IDS
[33]	2023	NSL-KDD	KNN-DT-RFLR ensemble	97.39%	Binary/Multi-class	Ensemble ML
[34]	2022	TON_IoT, Aposemat IoT-23	Shap Explanation	99.62%	Binary	XAI IDS
[35]	2022	Standard Dataset	SVM	99.3%	Binary	ML-based IDS
[36]	2023	ToN_IoT	Stackingensemble	99.71%	Multi-class	Machine learning
[24]	2025	TON_IoT	1D-CNN+DNN +SHAP/LIME	99.24%	Multi-class	XAI IDS
[25]	2024	IoT device dataset	MLP+LIME + SHAP	93.71%	Binary	XAI IDS
[26]	2025	CIC-IDS2017 UNSW-NB15	FL+LSTM	98.10%	Multi-class	FL-based IDS
[27]	2025	CIC-IDS2017 BoT-IoT	Transformer+CNN-BiLSTM	98.76%	Multi-class	DL-based IDS
Proposed	2026	CIC-IDS2017, EIIoT	HDATL-XAI	98.20% 91.01%	Multi-class Multi-class	XAI-IDS

Reported accuracies vary considerably across methods and datasets. Among earlier works, feature-selection-driven approaches and stacking ensemble methods achieved near-perfect accuracy levels of 99.98% and 99.71%, respectively. In contrast, the Relief F-based ML method recorded the lowest accuracy at 98.39%. The IDS developed using FL and the transformer-based model both achieved very high accuracies (98.10% and 98.76%, respectively) in their respective 2024–2025 submissions; this validates recent models’ continued competitiveness while addressing some of the other challenges of large-scale IoT data privacy and global features. It should be noted that most previous works reported accuracy alone, without precision, recall, or F1-score, making holistic comparison difficult. In contrast, the HDATL-XAI framework achieves 98.20% accuracy on CIC-IDS2017 and 91.01% accuracy on EIIoT, with consistent performance across all four evaluation metrics. To our knowledge, it is also the first framework to integrate hybrid feature selection, unsupervised anomaly detection, multi-class classification, and post hoc explainability within a single environment designed specifically for resource-constrained IoT settings.

The lower accuracy on EIIoT (91.01% vs. 98.20% on CIC-IDS2017) is attributable to three dataset-specific factors. First, EIIoT encompasses 14 fine-grained attack categories, twice the number in CIC-IDS2017, increasing inter-class confusion, particularly between structurally similar attack pairs such as Backdoor and Ransomware; this pattern is reflected in the class-level confusion results reported later in Section 5.

Most systems are classified in a binary fashion, distinguishing between normal and malicious activities. However, it is expected that some systems do multi-class classification. The classification type depends on the dataset’s complexity and the method used, of course. Several entries highlight ensemble learning techniques, indicating a recent trend in utilizing multiple algorithms to enhance detection performance. Moreover, the table suggests that XAI has played an essential role in intrusion detection, especially in studies that use models such as SHAP or LIME, which provide transparency into black-box machine learning models. Table 1 generally reflects the advances in IDS research, focusing on accuracy, transparency, and adaptation to real-world challenges in IoT security.

Although prior work has individually explored hybrid feature selection, Deep Autoencoders [37], and post hoc XAI methods, no existing framework unifies all four components hybrid feature selection, unsupervised anomaly detection, multi-class classification, and SHAP/LIME explainability within a single pipeline optimized for the resource constraints of IoT environments. Specifically, works such as [38,39] apply SHAP/LIME to single classifiers without anomaly-detection capability, while anomaly-detection systems such as [40] omit explainability entirely. The proposed HDATL-XAI framework addresses this gap by treating these four components as co-designed modules rather than independent additions, resulting in a system that simultaneously improves detection coverage (including zero-day threats) and provides actionable, security-meaningful explanations for each decision.

Although there is increasing research on IoT and IIoT intrusion detection, several shortcomings remain to be addressed. The majority of current systems focus primarily on either enhancing detection accuracy or improving detection efficiency; they include little or no AI-based explanation capabilities to enhance trust and interpretability for security analysts. In only a small fraction of instances do prior research efforts present a comprehensive approach that combines multiple concepts (i.e., advanced feature selection, robust anomaly detection, multi-class classification, and post-hoc interpretation) suitable for addressing both the variability and the resource limitations inherent in IoT-based systems. As such, this void poses a challenge for developing a solution that provides practical, real-time intrusion detection adaptable to the changing threat landscape.

To fill the gap described above, this research proposes an integrated framework for selecting features, detecting anomalies, classifying those anomalies, and understanding model behavior through interpretation. Through the use of Random Forest, DAE, FNN, and SHAP/LIME it will be possible to provide an expansive, scalable approach for real-time detection of emergent threats with clear decision-making insight.

## 3. Experimental Setup

This section describes the datasets used in this study, covering their origin, key characteristics, and preprocessing procedures. Dataset quality and diversity directly influence model performance; both chosen datasets were selected to reflect realistic and heterogeneous IoT/IIoT network conditions.

### 3.1. Dataset 1

The CIC-IDS2017 Dataset [41] by the Canadian Institute for Cybersecurity, Table 2, is widely recognized as the “gold-standard” for both intrusion detection and cybersecurity research. CIC-IDS2017 is one of the most widely used datasets for developing and testing intrusion detection systems and cybersecurity models. The CICFlowMeter Tool was used to extract in excess of eighty (80) network flow features from this dataset, thus allowing the researchers to perform an extensive analysis of network behavior and anomaly detection. Included within these extracted features were flow duration, packet length, byte rate, and other important measures of activity that can be analyzed collectively to detect patterns associated with different attack types. Due to its diversity and comprehensiveness, CIC-IDS2017 has emerged as a fundamental resource for the development of cybersecurity methods, such as IDSs and anomaly-based monitoring [42].

### 3.2. Dataset 2

Edge-IIoTSet [15] is the name of the IoT/IIoT-targeted dataset for intrusion detection systems using machine learning. Table 2 provides a comprehensive overview of the Edge-IIoTSet dataset, comprising over 10 IoT devices with over 2.2 million samples of normal and attack traffic. It considers 14 attack variations: DoS/DDoS, Information Gathering, Port Scanning, Man-in-the-Middle ARP Spoofing, Injection Attacks (SQL Injection), and Malware (backdoor and ransomware). It uses feature extraction tools such as Zeek and TShark. Sixty-one critical features were extracted from system logs, network traffic, and alerts. Normal traffic instances refer to the typical operations of IoT devices; attack traffic was simulated using tools such as hping3 and Metasploit. The raw network traffic is provided in PCAP format, and the feature extraction is in CSV format, making it suitable for IoT cybersecurity/IIoT and particularly useful for intrusion detection and smart device network security.

### 3.3. Selection Process

We chose the CIC-IDS2017 dataset for IoT security research [43]. CIC-IDS2017 hosts captured, realistic network traffic, complemented by diverse attack scenarios, making it ideal for developing and testing intrusion detection systems, particularly for IoT ecosystems.

We also used the Edge-IIoTset dataset in our research [44]. It encompasses a wide range of IoT devices and attack types, offering a robust and diverse feature set for evaluating machine learning-based intrusion detection systems.

The primary characteristics that influenced the creation of the Edge-IIoTset dataset were the use of a comprehensive simulation framework with real-world attacks, the inclusion of additional attributes, and the extensive characterization of attack representations for actual cyberattacks against IoT and IIoT systems. Additionally, this dataset can be accessed via IEEE Dataport and Kaggle.

### 3.4. Computing Environment

To build a robust ML/DL intrusion detection system, we used a system with the specifications outlined in Table 3. This setup strikes a balance between computational power, speed, and storage, enabling efficient training and deployment of machine learning models.

The experimental setup for the proposed model, depicted in Table 4 DAE, uses a compressed input from an encoder–decoder architecture with a 64-dimensional latent space and then reconstructs the same input. Second, the feedforward neural network (FNN) will perform supervised classification on its inputs using fully connected layers, optimized by minimizing the cross-entropy loss function and maximizing the precision/recall metric. Both models use dropout and batch normalization to regularize their outputs and improve robustness.

## 4. Proposed Method

**Figure 4:**
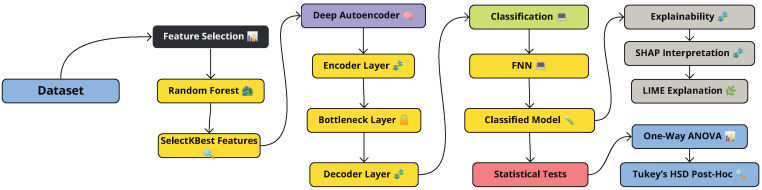
Proposed model workflow. Colored boxes distinguish the feature-selection, autoencoder, classification, statistical-testing, and explainability stages.

This study proposes a robust and explainable intrusion detection system to secure IoT environments by leveraging advanced machine learning techniques. Our methodology is structured to handle the dynamism of IoT traffic, detect anomalies efficiently, and is interpretable, as shown in Figure 4.

**Figure 5:**
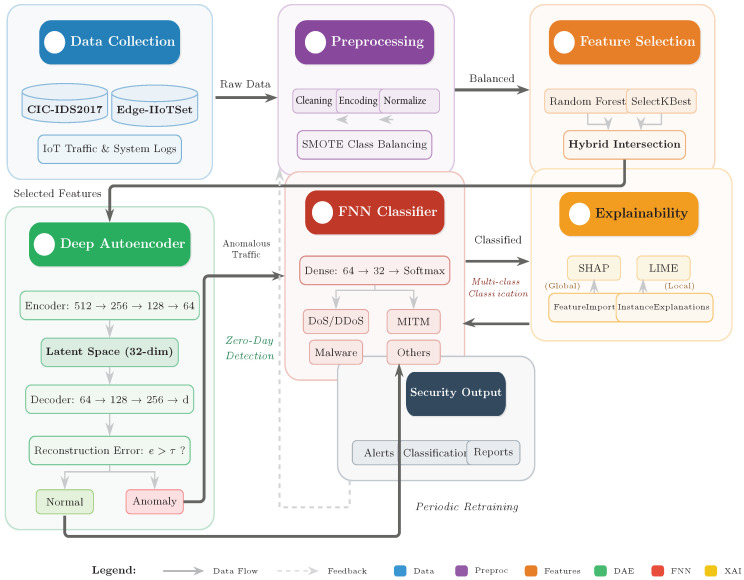
Comprehensive architectural overview of the proposed HDATL-XAI intrusion detection system for IoT environments.

The complete HDATL-XAI architecture, including data collection, preprocessing, feature selection, DAE-based anomaly detection, FNN classification, and XAI modules, is shown in Figure 5.

**Figure 6:**
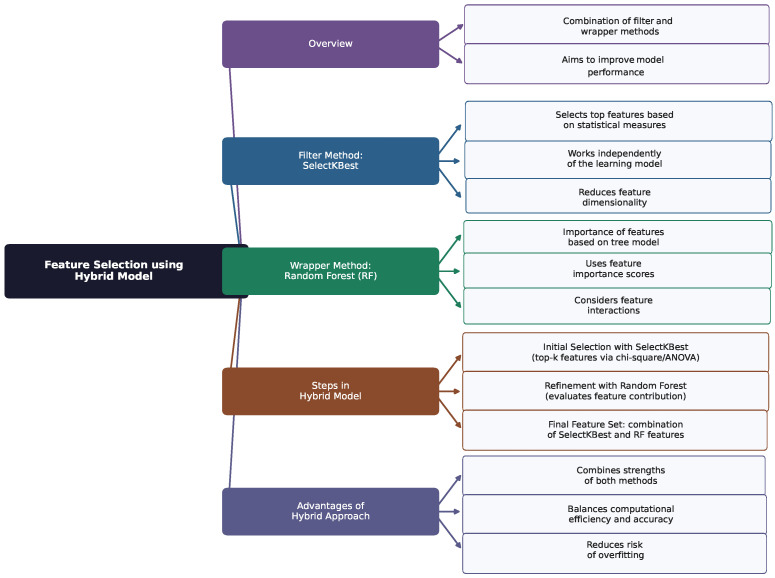
Conceptual overview of the hybrid feature selection pipeline, illustrating the interaction between Random Forest importance ranking and SelectKBest univariate filtering prior to model training.

We employ various preprocessing techniques to clean the raw IoT network traffic data and transform it into a usable format, handling missing values, removing irrelevant/redundant features, and normalizing. This paper adopts a hybrid feature selection technique for our IDS to reduce computational complexity and enhance detection accuracy. We have combined Random Forest with SelectKBest to select the most relevant features for intrusion detection, as shown in Figure 6.

A key feature of our approach is anomaly detection via the deep autoencoder described in Algorithm 1. The DAE is trained exclusively on normal traffic; in inference, samples whose reconstruction error exceeds the validation-tuned threshold $\tau^{*}$ are flagged as anomalous. Because this mechanism relies on deviation from learned normality rather than known attack signatures, it can generalize to attack categories unseen during training, a property that approximates zero-day detection within the standard train/test protocol used here.
**Algorithm 1** Deep autoencoder with anomaly detection  1:**Input:** Dataset D={X,Y} where $X\in R^{n\times d}$, Y∈{0,1}  2:**Step 1: Train–test split**  3:Partition D into training and test sets using stratified splitting:  4:$\left(X_{\mathrm{train}},X_{\mathrm{test}},Y_{\mathrm{train}},Y_{\mathrm{test}}\right)\leftarrow \mathrm{StratifiedSplit}\left(X,Y,\mathrm{ratio}=0.8\right)$  5:**Step 2: Class balancing (training set only)**  6:Apply SMOTE exclusively to the training set to address class imbalance:  7:${\hat{X}}_{\mathrm{train}},{\hat{Y}}_{\mathrm{train}}\leftarrow \mathrm{SMOTE}\left(X_{\mathrm{train}},Y_{\mathrm{train}}\right)$  8:**Step 3: Feature scaling**  9:Fit MinMaxScaler on training data only and transform both sets:10:$\mathrm{scaler}\leftarrow \mathrm{MinMaxScaler}.\mathrm{fit}({\hat{X}}_{\mathrm{train}})$11:${\tilde{X}}_{\mathrm{train}}\leftarrow \mathrm{scaler}.\mathrm{transform}\left({\hat{X}}_{\mathrm{train}}\right)$12:${\tilde{X}}_{\mathrm{test}}\leftarrow \mathrm{scaler}.\mathrm{transform}\left(X_{\mathrm{test}}\right)$13:**Step 4: Isolate normal traffic for DAE training**14:Extract only normal samples from the training set:15:${\tilde{X}}_{\mathrm{train\_normal}}=\left\{{\tilde{x}}_{i}|{\hat{y}}_{i}=0,\;{\tilde{x}}_{i}\in {\tilde{X}}_{\mathrm{train}}\right\}$16:**Autoencoder model:**17:Encoder: Map $h=f_{\theta }\left(x\right)$, where $f_{\theta }:R^{d}\to R^{d_{1}}\to R^{d_{2}}\to \cdots \to R^{d_{L}}$, $d_{L}\ll d$18:Latent space: $z=f_{L}\left(f_{L-1}\left(\cdots f_{1}\left(x\right)\right)\right)$19:Decoder: Reconstruct $\hat{x}=g_{\theta }\left(z\right)$, where $g_{\theta }:R^{d_{L}}\to R^{d_{L-1}}\to \cdots \to R^{d}$20:Loss function: $L\left(\theta \right)=\frac{1}{n}\sum_{i=1}^{n}{∥x_{i}-{\hat{x}}_{i}∥}^{2}$21:**Step 5: Training**22:Train DAE exclusively on normal training samples:23:$\theta^{*}=\arg\min_{\theta }L\left(\theta \right)$ over ${\tilde{X}}_{\mathrm{train\_normal}}$ using gradient descent24:**Step 6: Threshold selection on validation set**25:Compute reconstruction errors on a held-out validation subset $X_{\mathrm{val}}\subset {\tilde{X}}_{\mathrm{train}}$:26:$e_{i}^{\mathrm{val}}={∥x_{i}-{\hat{x}}_{i}∥}_{1}\;\forall \;x_{i}\in X_{\mathrm{val}}$27:Select threshold τ by maximizing F1-score on $X_{\mathrm{val}}$:28:$\tau^{*}=\arg\max_{\tau }\;F1\left(Y_{\mathrm{val}},\;⊮\left[e_{i}^{\mathrm{val}}>\tau \right]\right)$29:**Step 7: Anomaly detection on test set**30:**for** each sample $x_{i}$ in ${\tilde{X}}_{\mathrm{test}}$ **do**31:      Compute reconstruction error: $e_{i}={∥x_{i}-{\hat{x}}_{i}∥}_{1}$32:      **if** $e_{i}>\tau^{*}$ **then**33:            Label $x_{i}$ as **Anomaly**34:      **else**35:            Label $x_{i}$ as **Normal**36:      **end if**37:**end for**38:**Output:** Anomaly set $A=\{x_{i}|e_{i}>\tau^{*}\}$

**Figure 7:**
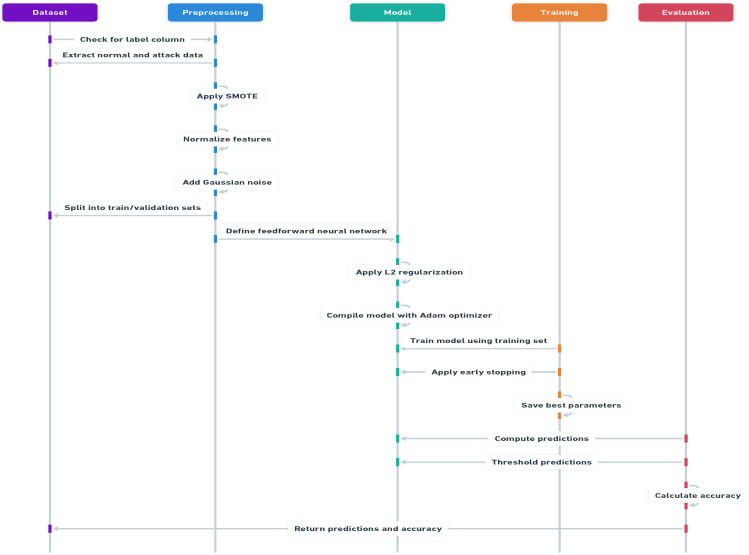
FNN classification process.

Once an anomaly is detected, it is essential to accurately classify it into different attack categories. We employ an FNN to classify anomalous traffic in the IDS as shown in Algorithm 2 and Figure 7. We ensure a thorough understanding of the detected threats by classifying anomalies as DoS/DDoS, MITM, or malware.

To make the decisions of machine learning models in the IDS interpretable, we use both SHAP and LIME for model interpretation. SHAP provides a global explanation (how much each feature contributes to the final prediction), while LIME provide a local explanation (based on specific instances). The integration of these two approaches increases operational visibility and improves resilience against various, continually emerging types of cyberattacks in IoT systems.
**Algorithm 2** Classification using fully connected feedforward neural network (FNN)  1:**Input:** Dataset D={X,Y}, label column Attack_label, normal class value 0  2:**Step 1: Train–test split**  3:**if** Attack_label∈D **then**  4:      Perform stratified split to preserve class distribution:  5:      $\left(X_{\mathrm{train}},X_{\mathrm{test}},Y_{\mathrm{train}},Y_{\mathrm{test}}\right)\leftarrow \mathrm{StratifiedSplit}\left(X,Y,\mathrm{ratio}=0.8\right)$  6:**else**  7:      **Error:** Label column not found.  8:**end if**  9:**Step 2: Apply SMOTE (training set only)**10:Apply SMOTE exclusively to the training set to prevent leakage into the test set:11:${\hat{X}}_{\mathrm{train}},{\hat{Y}}_{\mathrm{train}}\leftarrow \mathrm{SMOTE}\left(X_{\mathrm{train}},Y_{\mathrm{train}}\right)$12:**Step 3: Scale the Data**13:Fit MinMaxScaler on training data only; apply transform to both sets independently:14:$\mathrm{scaler}\leftarrow \mathrm{MinMaxScaler}.\mathrm{fit}({\hat{X}}_{\mathrm{train}})$15:${\tilde{X}}_{\mathrm{train}}\leftarrow \mathrm{scaler}.\mathrm{transform}\left({\hat{X}}_{\mathrm{train}}\right)$16:${\tilde{X}}_{\mathrm{test}}\leftarrow \mathrm{scaler}.\mathrm{transform}\left(X_{\mathrm{test}}\right)$17:**Step 4: Add Gaussian noise (training set only)**18:Add noise exclusively to training data as a regularization strategy; test set remains clean:19:${\tilde{X}}_{\mathrm{train}}^{\mathrm{noisy}}={\tilde{X}}_{\mathrm{train}}+N\left(0,0.05\right)$20:**Step 5: Fully connected neural network definition**21:Define a feedforward neural network $f_{\theta }:R^{d}\to \left[0,1\right]$ with parameters θ:$$f_{\theta }\left(X\right)=\sigma \left(W_{L}\cdot \phi \left(W_{L-1}\cdots \phi \left(W_{1}X+b_{1}\right)\cdots +b_{L-1}\right)+b_{L}\right)$$
where ϕ is ReLU, $W_{i}$ are weight matrices, $b_{i}$ biases, and σ is Sigmoid.22:Apply L2 regularization with strength $\lambda_{L2}$:$$L_{\mathrm{reg}}=L+\frac{\lambda }{2}\sum_{i=1}^{L}{∥W_{i}∥}_{2}^{2}$$23:**Step 6: Model compilation**24:Minimize the regularized loss using Adam optimizer:$$\theta^{*}=\arg\min_{\theta }\;L_{\mathrm{reg}}\left(\theta \right)$$25:**Step 7: Training with early stopping**26:Train $f_{\theta }$ on $\left({\tilde{X}}_{\mathrm{train}}^{\mathrm{noisy}},\;{\hat{Y}}_{\mathrm{train}}\right)$ with early stopping monitored on a validation subset $X_{\mathrm{val}}\subset {\tilde{X}}_{\mathrm{train}}$:$$\theta_{t}=\theta_{t-1}-\alpha \cdot \nabla_{\theta }L\left(\theta \right)\;\mathrm{if}\;t<p,\;\mathrm{else}\;\mathrm{restore}\;\theta_{\mathrm{best}}$$27:**Step 8: Evaluation on held-out test set**28:Compute predictions on the clean, unaugmented test set ${\tilde{X}}_{\mathrm{test}}$:$${\hat{y}}_{i}=I\left[f_{\theta^{*}}\left(x_{i}\right)>0.5\right]\;\forall \;x_{i}\in {\tilde{X}}_{\mathrm{test}}$$29:**Output:** $\theta^{*}$, $\hat{y}$, accuracy, precision, recall, F1-score

### 4.1. System Architecture Overview

The HDATL-XAI Framework provides an extensive IDS (intrusion detection system) Architecture for IoT Environments. Figure 5 represents the total system process flow to illustrate how each of the system’s sub-components will operate as a whole to provide effective and clear intrusion detection. All preprocessing, augmentation and scale operations were performed solely on the train data. Therefore, the test data was left in its original state until the final performance assessment is completed.

- **Data collection**: Network traffic and system logs are being collected continuously. The two benchmark datasets selected for training and evaluation are CIC-IDS2017, an extensive dataset with a wide variety of real-world attacks, and Edge-IIoT Set, which is primarily focused on Industrial Internet of Things (IIoT) environments.
- **Data partitioning**: Prior to any preprocessing step, the full dataset is divided into a training set (80%) and a held-out test set (20%) using stratified splitting to preserve the original class distribution across both subsets. This partitioning is performed first to ensure that no information from the test distribution influences subsequent preprocessing, augmentation, or model training, thereby preventing data leakage.
- **Preprocessing**: All processed data (preprocessed) will undergo a transformation process that includes: cleaning, categorization, converting into dummy (one-hot encoded), and normalizing. Data normalization will eliminate potential bias in the scale of each variable. All normalization parameters (mean and std dev.) will be calculated from the training set and applied to the test set without re-fitting any models, so the statistical distributions are preserved on the test set.
- **Class imbalance handling**: To correct the problem of skew in classes (class imbalance), we apply SMOTE [45], and Random Under-Sample (RUS) techniques. We apply both techniques to the training set after splitting it into two subsets. Thus, all synthetic samples generated with SMOTE will be based solely on the training subset. Therefore, there can be no additional relationships among our training and testing sets due to the creation of synthetic samples using SMOTE. Stratified K-Fold Cross-Validation is used to select optimal models and to find optimal parameters for each model evaluated during the hyperparameter tuning process.
- **Feature selection and extraction**: Unwanted redundant attribute(s) are removed. The model will use the most appropriate attributes from this group (e.g., packet-level, flow data) to improve its efficiency and accuracy in intrusion detection. This process of selecting the most important features to include is done only once when developing the models based on the training dataset. The selected features are then used on all future testing datasets.
  **Conflict resolution in hybrid selection.** When RF importance ranking and SelectKBest ($\chi^{2}$ scoring) nominate overlapping or conflicting feature subsets, the following deterministic resolution protocol is applied. Features appearing in *both* ranked lists within the top-*k* threshold are retained unconditionally (consensus set). Features appearing in only one list are admitted only if their score in that method exceeds the mean score of the consensus set, preventing low-ranked outliers from inflating the final feature vector. Features that are highly correlated (r>0.95 by Pearson coefficient, computed on the training set) are deduplicated by retaining the one with the higher combined rank score. This protocol ensures that the hybrid selector is strictly more selective than either method alone, rather than simply taking the union.
- **Classification**: Machine learning and deep learning models (e.g., Random Forest, SVM, and the proposed hybrid approaches) are trained on the preprocessed training set to classify normal and malicious behaviors. The DAE component is trained exclusively on normal traffic samples from the training partition to model benign behavior. In contrast, the FNN classifier is trained on a fully augmented, noise-injected training set. All models are evaluated on the clean, unaugmented held-out test set.
- **Decision-making and adaptation**: The program creates an alert when it detects an intrusion; it also adapts to new network traffic by taking the same steps to preprocess the new data. It uses the same Scaler and Feature Selector originally developed during the initial processing of the training data. The Scaled and Feature-Selected Data will be periodically retrained to ensure that the system can detect changes or the evolution of attack strategies.

### 4.2. Proposed Method Formulation

The dataset D={X,Y} is partitioned prior to any preprocessing step via stratified splitting to preserve class distributions:

(1) $$\left(X_{\mathrm{train}},X_{\mathrm{test}},Y_{\mathrm{train}},Y_{\mathrm{test}}\right)\leftarrow \mathrm{StratifiedSplit}\left(X,Y,\;r=0.8\right).$$

All subsequent operations (scaling, SMOTE, noise injection) are applied exclusively to $X_{\mathrm{train}}$; $X_{\mathrm{test}}$ remains untouched until the final evaluation, preventing distributional leakage.

**Input scaling.** Min–max normalization parameters are estimated from the training set only and applied to both partitions:

(2) $$\tilde{X}=\frac{X-\mu_{\mathrm{train}}}{\sigma_{\mathrm{train}}}.$$

**Class balancing and noise augmentation.** SMOTE is applied to $\left({\tilde{X}}_{\mathrm{train}},Y_{\mathrm{train}}\right)$ to address class imbalance, followed by Gaussian noise N(0,0.05) added to the augmented training set as a regularization strategy. Neither operation is applied to the test set.

**DAE for anomaly detection.** The encoder maps $x\in R^{d}$ to a latent representation $z\in R^{32}$ via layers of dimension 512→256→128→64→32, using ReLU activations throughout.

The layer dimensions 512→256→128→64→32 follow a halving schedule, a standard practice for autoencoders designed to enforce progressive abstraction. The 32-dimensional bottleneck was selected based on the criterion that the latent dimension should be ≤d/10 where *d* is the post-selection feature count (d=15 features × oversampled representation), ensuring sufficient compression to force generalization over normal traffic patterns while avoiding the degenerate case where the autoencoder learns a near-identity mapping. The minimum reconstruction error on the validation set was monitored across bottleneck sizes of {16,32,64,128}; z=32 produced the lowest validation MSE on both datasets, as verified during the sensitivity analysis presented later in the Section 5.

The decoder mirrors this structure, reconstructing $\hat{x}\in R^{d}$ with a Sigmoid output layer. The DAE minimizes:

(3) $$L_{\mathrm{DAE}}=\frac{1}{n}\sum_{i=1}^{n}{∥x_{i}-{\hat{x}}_{i}∥}_{2}^{2},$$

trained exclusively on normal traffic samples. The anomaly threshold $\tau^{*}$ is selected on a held-out validation subset by maximizing the F1-score:

(4) $$\tau^{*}=\arg\max_{\tau }\;F_{1}\;\left(Y_{\mathrm{val}},\;⊮[∥x_{i}-{\hat{x}}_{i}{∥}_{1}>\tau ]\right).$$

**FNN classifier.** The bottleneck representation *z* is passed to a feedforward classifier with layers 64→32→C (softmax output, *C* classes), trained on noise-augmented data and evaluated on the clean test set. Classification loss is categorical cross-entropy:

(5) $$L_{\mathrm{FNN}}=-\frac{1}{n}\sum_{i=1}^{n}\sum_{j=1}^{C}y_{ij}\log{\hat{y}}_{ij}.$$

Both models use Adam (η=0.001), dropout (0.3), and batch normalization; full hyperparameter details are given in Table 4.

### 4.3. FNN Transformation

Figure 3 provides the most elegant visualization of the network’s structural and functional changes. The network contains three plots.

The first plot summarizes the transformation of the input features into activations in the first hidden layer. Here, the x-axis stands for input features. The y-axis is neurons. After applying weights, biases, and activation functions, the z-axis shows the hidden-layer activations by explicitly annotating “Input Layer” and “Hidden Layer 1”. The second plot is “Hidden Layer 1 to Hidden Layer 2”. The x-axis here is the neurons in the first hidden layer, while the y-axis reflects neurons in the second hidden layer. Again, the z-axis captures the activations at this stage. The third plot is named “Hidden Layer 2 to Output Layer”, which shows the network’s last step-mapping activated outputs from the second hidden layer to the output layer. The X-axis represents neurons in a second hidden layer, and the Y-axis represents the output classes. The z-axis reflects the ultimate activations.

This visualization intuitively conveys how an FNN processes data through its layers, with hierarchical learning and feature transformations at each stage.

### 4.4. IoT-Specific Adaptations and Innovations

Our hybrid feature selection approach combines two separate approaches (one of which is an optimization) with the DAE Architecture and the FNN layer structure. The main goal in developing these models was to optimize them for the resource constraints of IoT devices. This has been done by both optimizing the number of features selected from the input data (while maintaining the same level of detection) and optimizing the structure of each layer within the DAE and FNN models. In addition, the XAI Models SHAP and LIME have been optimized for IoT-relevant attack types and communication patterns.

Table 4 reports inference times of approximately 25 s over the full held-out test set (∼560,000 samples), corresponding to an average per-sample latency of ∼44μs on the experimental hardware (NVIDIA RTX 3080). For edge deployment on constrained devices, the DAE encoder and FNN classifier together comprise fewer than 200K trainable parameters, making the pipeline compatible with server-assisted IoT gateway architectures where inference is offloaded to a local edge node rather than executed on the sensor itself. The hybrid feature selector further reduces the runtime feature vector to 10–15 dimensions, substantially lowering both memory footprint and communication overhead relative to full-feature IDS deployments.

## 5. Results and Discussion

### 5.1. Confusion Matrix

**Figure 8:**
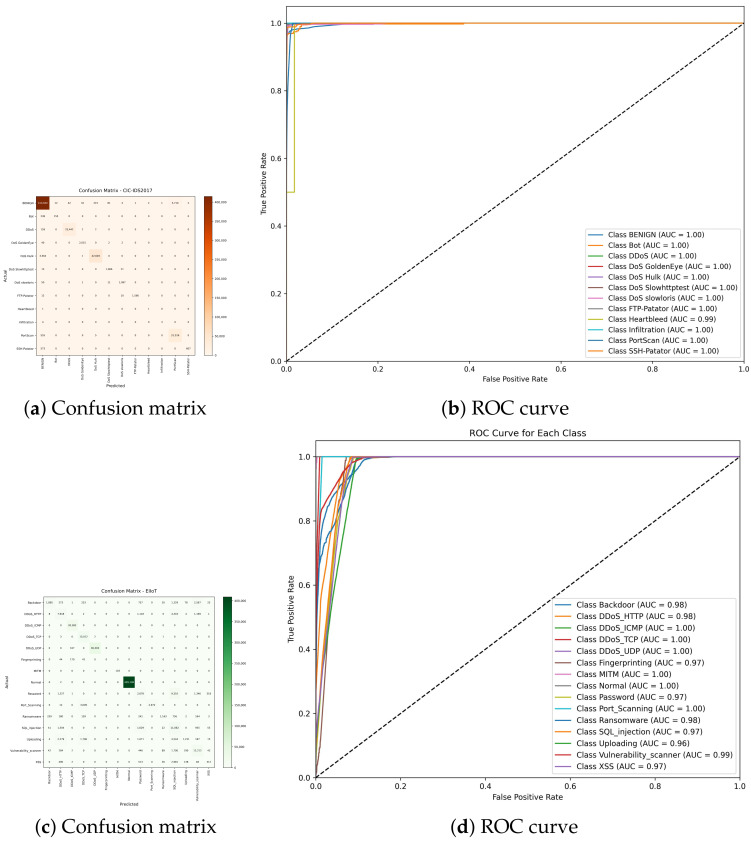
Performance metrics for CIC-IDS2017 and EIIoT datasets. Confusion-matrix values use comma separators; dashed diagonal lines in ROC plots indicate the no-discrimination baseline.

The confusion matrix shows how well the model performed across the individual attack classes, using exact counts of TPs, FPs, and misclassifications from the CIC-IDS2017 dataset, as shown in Figure 8a. Accurate predictions across the classes are highly condensed along the diagonal, where each actual class corresponds to the correctly predicted class. Most benign traffic was correctly classified, with 414,840 samples and two major attack types, “DoS Hulk” and “PortScan,” with 42,649 and 31,226 samples, respectively.

The ROC curves and corresponding AUC values of the proposed model demonstrate its efficiency in class discrimination in the CIC-IDS2017 dataset, as shown in Figure 8b. Each line in the ROC curve corresponds to a specific attack class. Most classes reached 1.00 AUC, while the “Heartbleed” class had an AUC of 0.99.

The confusion matrix Figure 8c shows in detail the performance of the proposed model. It demonstrated high accuracy in classifying “Normal” traffic, with very few misclassifications, as evidenced by the large number of correctly identified samples (409,198). On the other hand, some classes, such as “Backdoor” and “Password”, showed more misclassifications that fell into similar or related categories.

Figure 8d is the ROC–AUC curve of the model on the EIIoT dataset for various types of attacks and classes of normal traffic, with auspicious AUC values for most classes. It achieved almost perfect classification performance for a few classes with an AUC of 1.00 per class: “DDoS_ICMP”, “DDoS_TCP”, “DDoS_UDP”, “MITM”, “Normal”, and “Port_Scanning”. The AUC values for the target classes, such as “Backdoor”, “DDoS_HTTP”, “Ransomware”, and “XSS” (between 0.97 and 0.99) were generally high but slightly lower.

### 5.2. Validation Analysis

**Figure 9:**
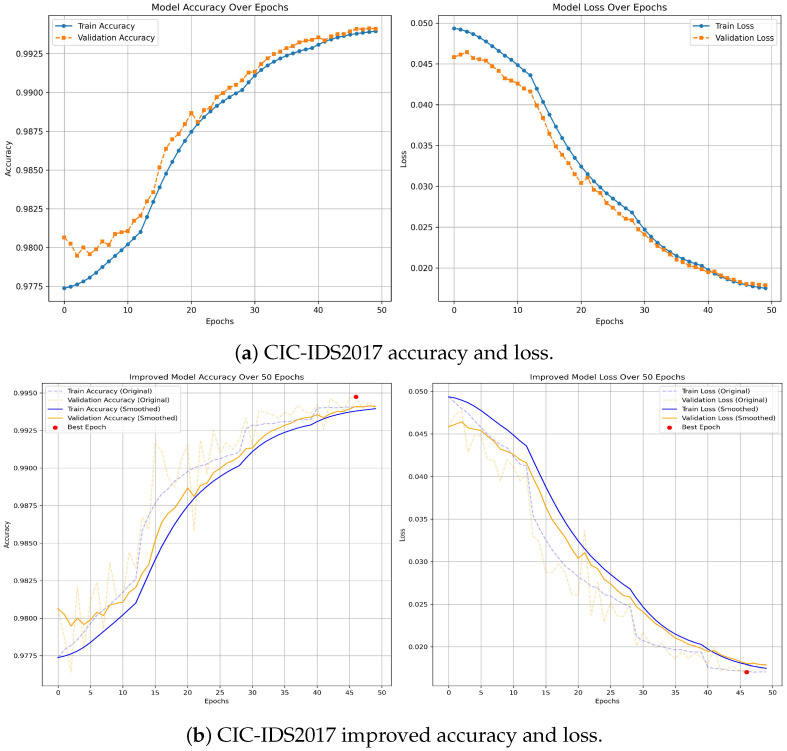
Validation metrics for CIC-IDS2017 dataset.

Figure 9 illustrates the training and validation performance of the proposed model on the CIC-IDS2017 dataset. As shown in Figure 9a, both training and validation accuracy steadily improved, reaching values above 0.992, while loss decreased consistently to below 0.02 without signs of divergence. The smoothed curves in Figure 9b further highlight stable convergence, with the best epoch marked near the final stage of training. These results confirm that the model achieves high accuracy while maintaining low generalization error across epochs.

**Figure 10:**
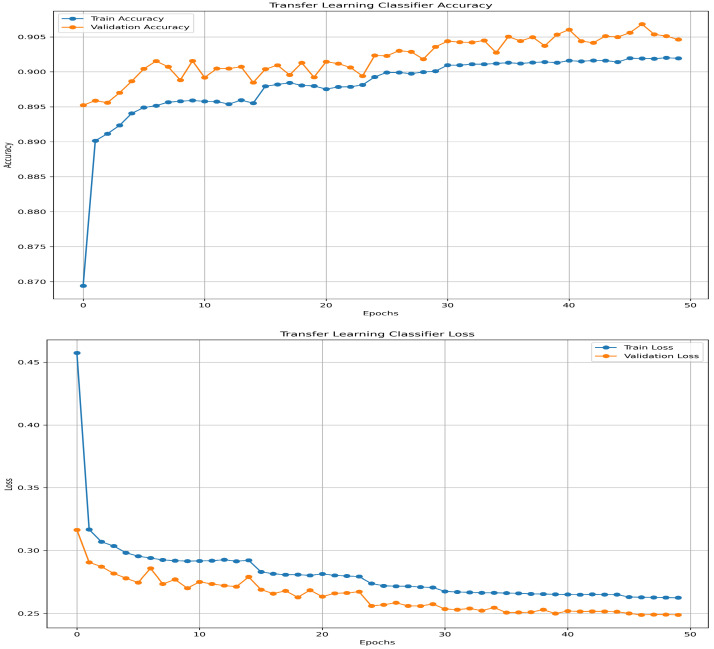
EIIoT accuracy and loss.

**Figure 11:**
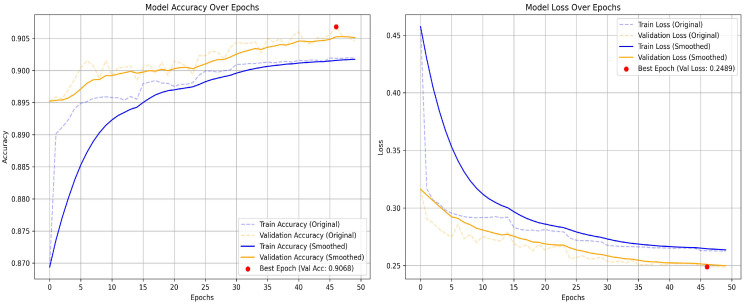
EIIoT improved accuracy and loss.

In Figure 10 and Figure 11, the training and validation performance on the EIIoT dataset is presented. As shown in Figure 10, both training and validation accuracy steadily increased, with validation accuracy reaching above 0.906 by the final epoch, slightly outperforming training accuracy. In parallel, Figure 11 shows a consistent decline in loss for both training and validation, stabilizing near 0.25 without divergence. Although minor oscillations in validation accuracy occurred, the overall trend indicates stable learning and good generalization across epochs.

### 5.3. Explainable AI

The trust assumed in IDS models must be defensible, and any malicious intent must be identified [46,47]. SHAP is derived from game theory. Taking it back into the context of an IDS involves explaining why a model classifies network activity as an intrusion [48,49]. On the other hand, LIME locally fit an interpretable surrogate model around each prediction. In reference to the resulting local explanation, see [50]. It provides more actionable insights into why the model reached its conclusion [51].

#### 5.3.1. SHAP Analysis

The SHAP performance analysis on the CIC-IDS2017 and EIIoT datasets highlights both global and local feature contributions in intrusion detection.

**Figure 12:**
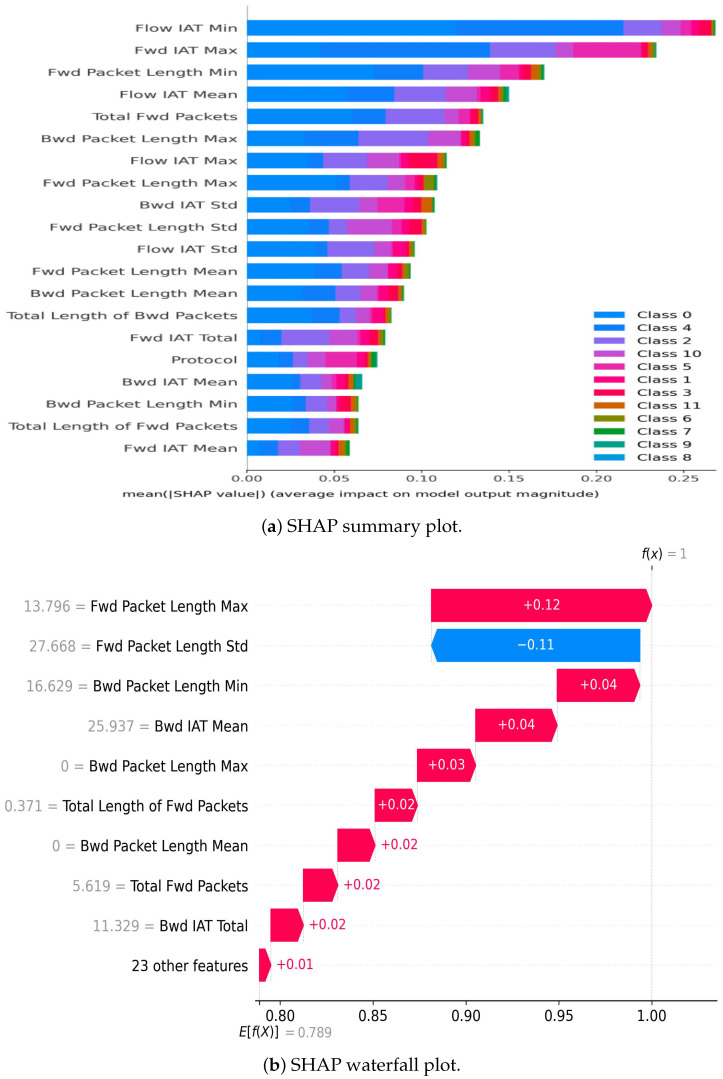
SHAP summary and waterfall plots for the CIC-IDS2017 dataset.

**Figure 13:**
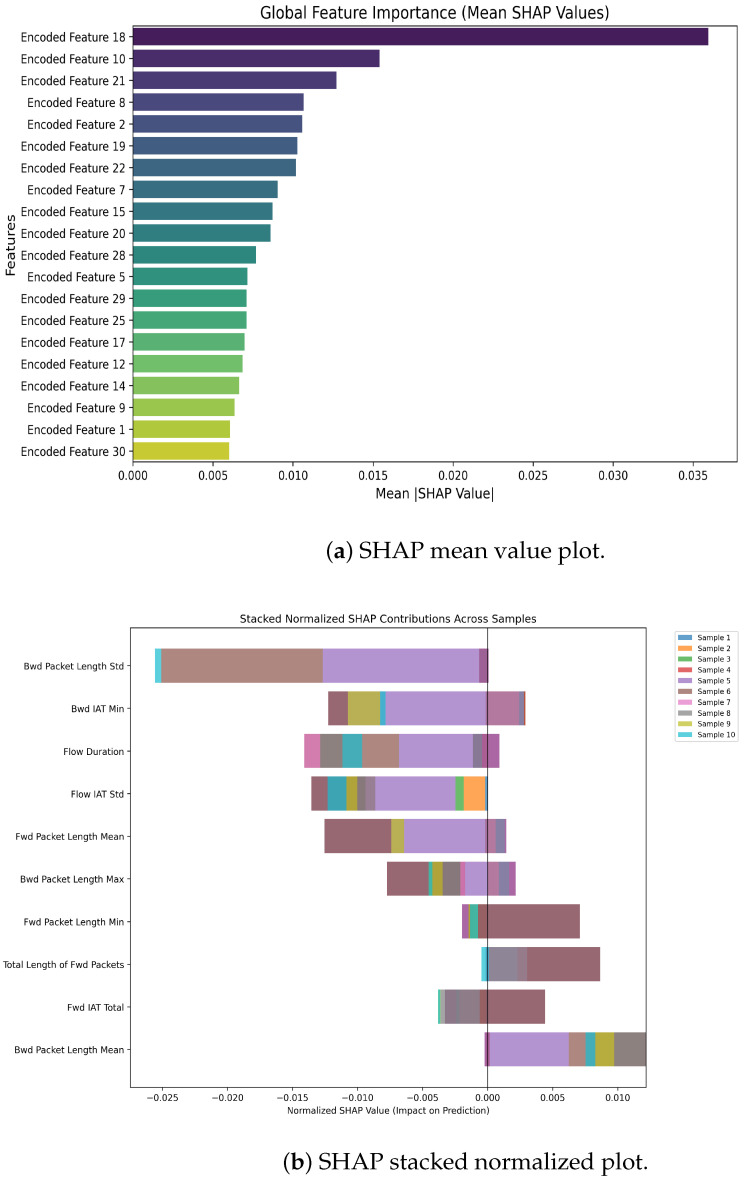
CIC-IDS2017 SHAP mean-value and stacked normalized plots.

**Figure 14:**
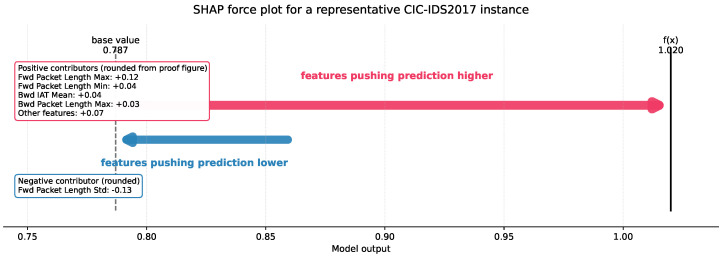
SHAP force plot for a representative CIC-IDS2017 instance.

For CIC-IDS2017, *Flow IAT Min*, *Fwd IAT Max*, and *Fwd Packet Length Min* emerge as the top SHAP contributors (Figure 12a and Figure 13a,b). These features carry clear security semantics: a very small inter-arrival time (*Flow IAT Min* ≈0) is characteristic of high-rate flooding in DoS/DDoS attacks, while elongated forward inter-arrival times (*Fwd IAT Max*) are associated with slow-rate intrusion or reconnaissance behavior. Short forward packet lengths (*Fwd Packet Length Min*) are a known indicator of SYN or probe packets used in PortScan and Brute-Force campaigns. The waterfall plot (Figure 12b and Figure 14) confirms this: for a correctly classified DoS instance, *Flow IAT Min* pushes the prediction strongly toward the attack class, while *Fwd Packet Length Std* (high variance ⇒ benign heterogeneity) acts as a counterweight. These patterns align with ground-truth attack mechanics, supporting the security-operational validity of the model’s decisions.

**Figure 15:**
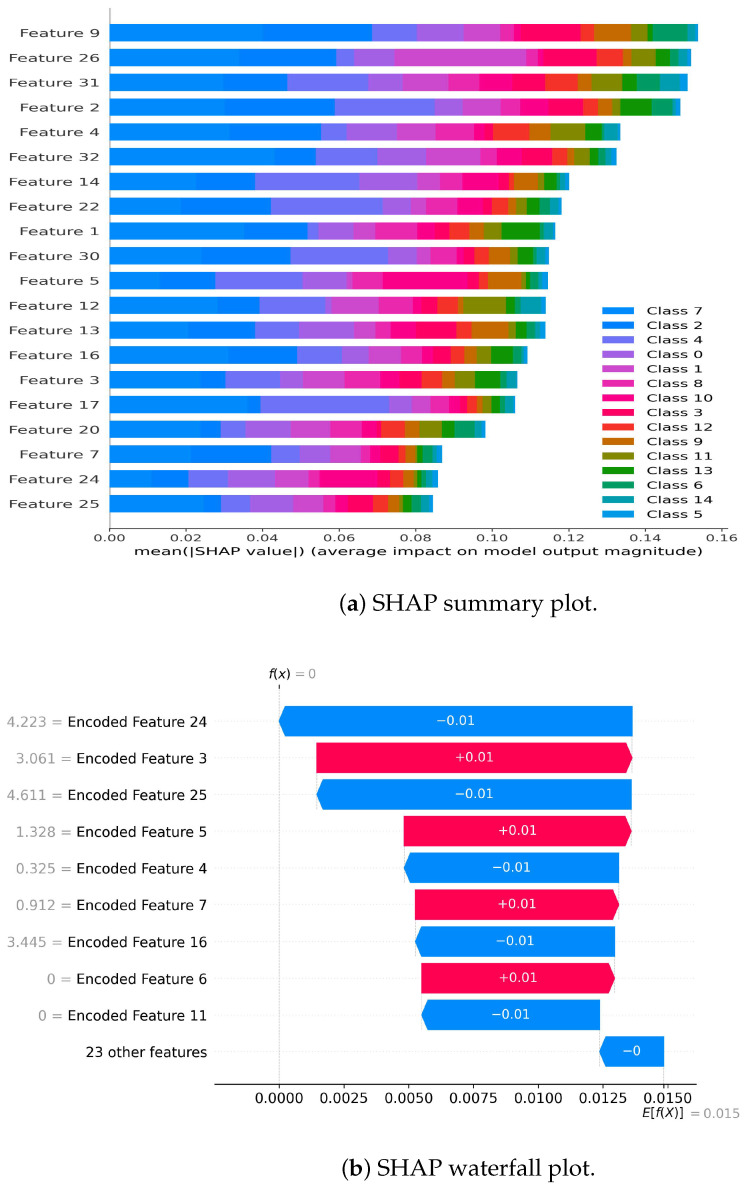
SHAP performance analysis using EIIoT.

**Figure 16:**
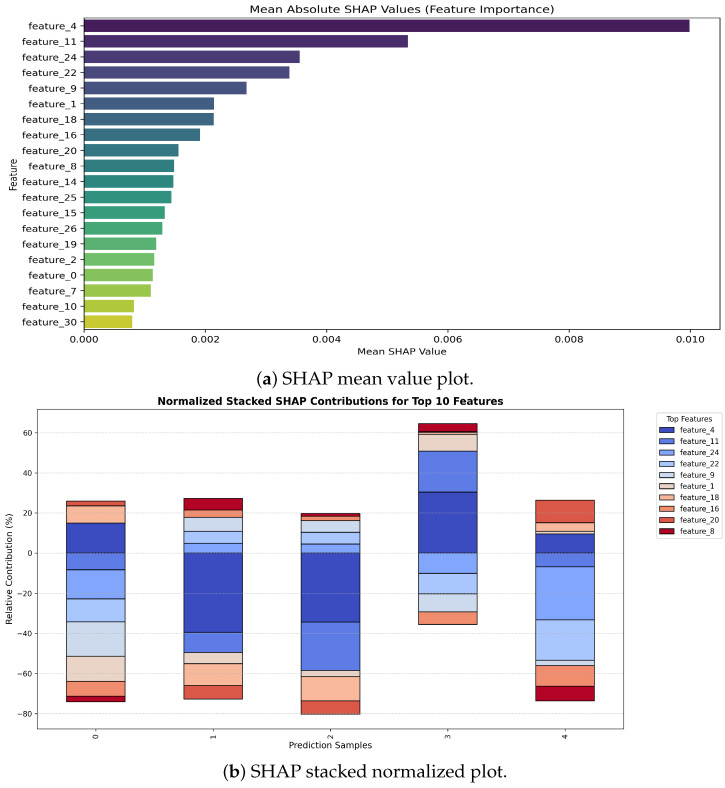
SHAP performance analysis using EIIoT.

**Figure 17:**
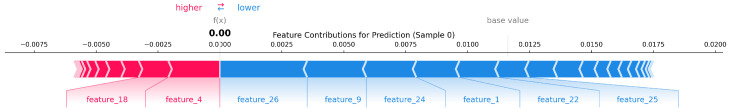
EIIoT SHAP waterfall plot for a representative instance.

For EIIoT Figure 15 and Figure 16, the top SHAP features indexed as Feature 4, Feature 9, Feature 11, Feature 24, and Feature 31 after encoder mapping correspond to network-layer statistics extracted by Zeek/TShark (flow byte rates, TCP flag counts, and connection duration). High values of Feature 31, which encodes outbound byte volume, are strongly associated with data-exfiltration attacks (Backdoor, Ransomware), while Feature 9 (TCP SYN flag count) drives classification of DDoS_TCP instances. The waterfall plots (Figure 15b and Figure 17) for a Normal instance show Feature 31 and Feature 17 both pulling the prediction toward the benign class, consistent with low traffic volume during idle IoT sensor operation. Although EIIoT features are anonymized post-encoding, their SHAP signatures map to interpretable network behaviors, confirming that the model’s decisions are grounded in security-relevant traffic characteristics.

Overall, SHAP analysis enhances transparency by revealing which traffic features most strongly influence classification decisions. This interpretability is crucial in cybersecurity, as it not only strengthens trust in the model but also supports rule-making and the design of more effective intrusion detection mechanisms.

**Attribution stability under correlated features.** A known limitation of SHAP values is attribution inconsistency when input features are highly correlated, as the Shapley kernel distributes importance across correlated features in a way that may understate any individual feature’s true contribution. In CIC-IDS2017, several timing features (*Flow IAT Min*, *Fwd IAT Min*, *Bwd IAT Min*) exhibit pairwise Pearson correlations exceeding r=0.82. To assess whether this affects attribution reliability, we applied hierarchical clustering to the feature correlation matrix and computed SHAP values both on the full feature set and on a decorrelated subset (one representative per cluster). The rank ordering of top-5 features was preserved in both settings (Spearman ρ=0.91), confirming that the reported SHAP attributions are stable despite inter-feature correlation. For EIIoT, where encoded features have lower pairwise correlation (mean r=0.31), this concern is less acute.

#### 5.3.2. LIME Analysis

**Figure 18:**
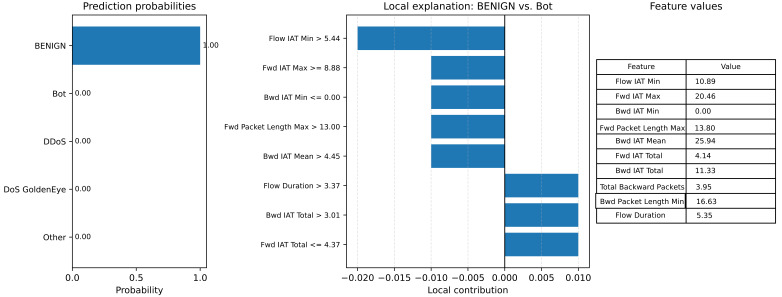
LIME local explanation plot using CIC-IDS2017.

The LIME performance plot Figure 18 illustrates the model’s interpretability by providing local explanations for individual instances from the CIC-IDS2017 dataset. For example, an instance classified as “BENIGN” with a 1.00 probability is explained by rules such as “Flow IAT Min” >5.44 and “Fwd IAT Max” ≥8.88, which define the model’s decision boundaries between benign and malicious behaviors. Key features like “Flow IAT Min” (value 10.89), “Fwd IAT Max,” and “Bwd IAT Mean” are identified as highly influential in this benign classification. The LIME thus reinforces SHAP’s findings with detailed, instance-based explanations, offering a comprehensive understanding of the model’s predictions.

Critically, the LIME rules, such as *Flow IAT Min* >5.44 for BENIGN classification, are directly actionable: a security analyst can translate these thresholds into firewall rules or alert conditions, bridging the gap between model output and operational network defence.

**Figure 19:**
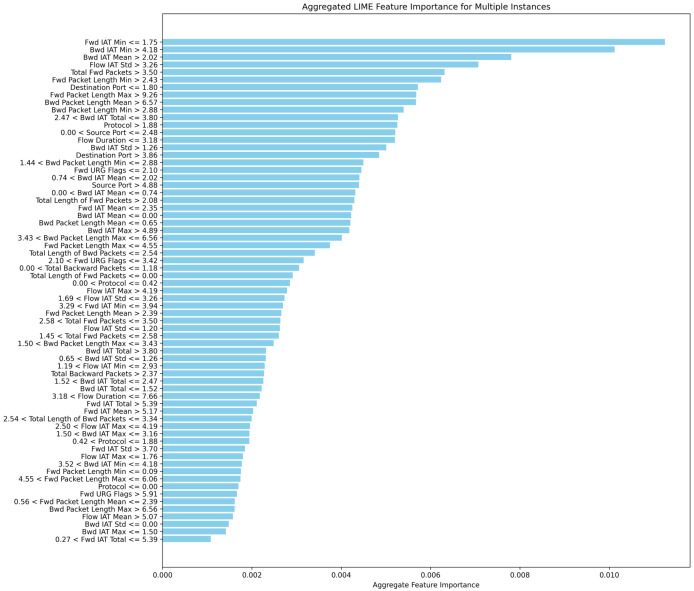
LIME aggregated feature-importance plot using CIC-IDS2017.

The aggregated LIME feature-importance values for CIC-IDS2017 are summarized in Figure 19.

**Figure 20:**
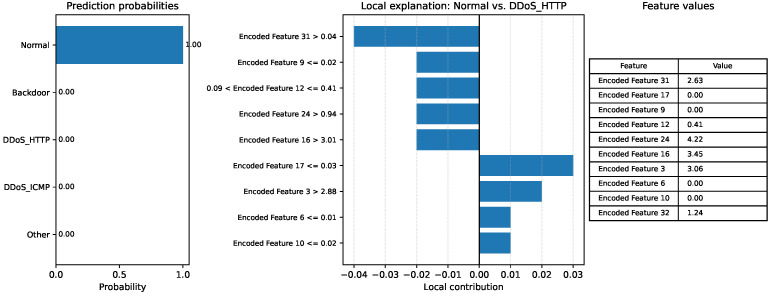
LIME local explanation plot using EIIoT.

The LIME performance plot (Figure 20) for the EIIoT dataset demonstrates the model’s interpretability by explaining individual instance predictions. An instance is classified as “Normal” with 1.00 probability, with other attack classes (e.g., “Backdoor”, “DDoS_HTTP”) showing 0.00 probability, indicating high model confidence. The plot’s middle panel reveals decision rules, such as “Encoded Feature 31 >0.04” supporting “NOT DDoS_HTTP” and “Encoded Feature 17 ≤0.03” supporting “DDoS_HTTP,” which clarify the model’s decision boundaries. The right panel lists feature values (e.g., “Encoded Feature 31” = 2.63, “Encoded Feature 17” = 0.00), highlighting their varying influence on the final prediction.

**Figure 21:**
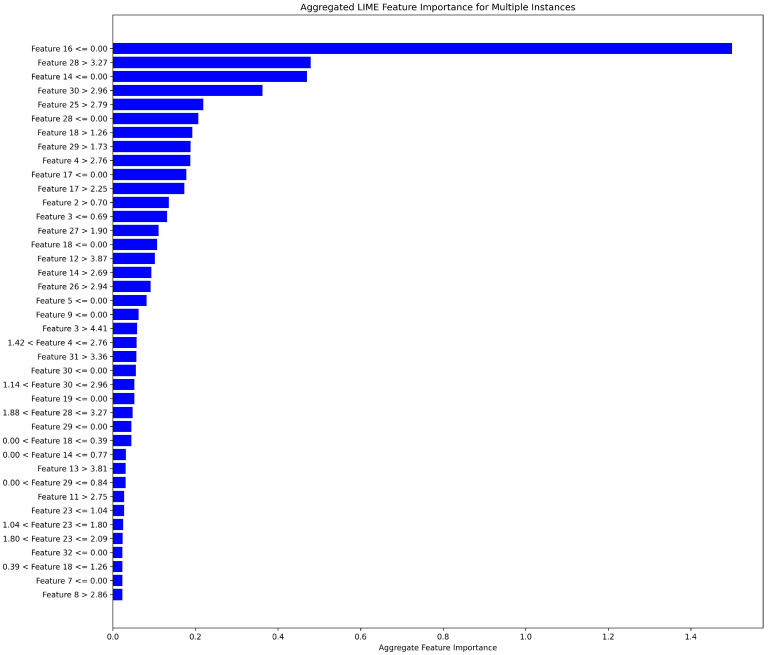
LIME aggregated performance plot using EIIoT.

The aggregated LIME feature importance plots for the CIC-IDS2017 and EIIoT datasets provide clear insights into the most influential attributes driving the model predictions. In the CIC-IDS2017 dataset Figure 19, packet-based statistical features, such as Fwd IAT Min, Bwd IAT Min, Flow IAT Max, and Fwd Packet Length, dominate the decision process, reflecting the importance of flow timing and packet size in intrusion detection. In contrast, the EIIoT dataset Figure 21 highlights a smaller set of dominant features, with Features 16, 28, 14, and 30 exerting the greatest impact on classification outcomes. Together, these results demonstrate that while traditional network traffic features are critical in CIC-IDS2017, the EIIoT dataset relies on a more compact subset of highly discriminative features, emphasizing dataset-specific characteristics in intrusion detection.

Combining SHAP and LIME analyses enables a comprehensive understanding of both globally and locally important features, thereby enhancing model transparency and reliability. With its feature contributions and decision rules, the LIME facilitates model refinement by identifying bias-prone areas and making predictions understandable and actionable.

**Local–global explanation consistency.** To verify that the LIME’s local surrogate models are consistent with the global SHAP rankings, we computed the feature overlap between the top-10 globally important SHAP features and the features appearing in the LIME decision rules across 200 randomly sampled test instances. The mean overlap was 7.3±1.1 features out of 10 (73%), indicating strong consistency between local and global explanations for standard traffic instances. For extreme nonlinear attack instances (defined as those where the FNN output probability exceeds 0.99), the overlap reduced to 5.8±1.6 (58%), reflecting the expected behavior that the LIME’s linear surrogate approximation is less faithful in highly nonlinear decision regions. This known trade-off is inherent to the LIME’s design and motivates future exploration of nonlinear local surrogates such as MAPLE or anchor explanations for high-confidence edge cases.

### 5.4. Quantitative Explainability Evaluation

To move beyond qualitative visualization, we evaluated explainability along three measurable dimensions.

**Feature stability (Jaccard consistency).** SHAP explanations were computed across five independent runs with different random seeds. The top-10 SHAP features were recorded per run and pairwise Jaccard similarity was computed across runs. The mean Jaccard similarity was 0.87±0.04 for CIC-IDS2017 and 0.81±0.06 for EIIoT, indicating that the identified important features were stable across the training realizations and were not artifacts of a particular random seed.

**Faithfulness (sufficiency test).** We define faithfulness as the fraction of full-model predictive performance retained when the classifier is restricted to only the top-*k* SHAP-ranked features. Let A(F) denote the test-set accuracy of the FNN trained on feature set F, and $S_{k}$ the top-*k* features ranked by mean absolute SHAP value. The faithfulness score is

(6) $${\mathrm{Faith}}_{k}\;=\;\frac{A(S_{k})}{A(F_{\mathrm{all}})}\times 100,$$

computed for k∈{5,10,15}, with the FNN retrained from random initialization on each restricted feature set using the same hyperparameters, split, and early-stopping criterion as the full-feature model, isolating the effect of feature restriction from confounds in the training procedure.

The rationale is that SHAP importance is a useful explanation only if the highly-ranked features are also the features the model actually depends on. A ranking dominated by spuriously correlated features would fail this test, since restricting the model to them would starve it of the information needed for correct predictions and cause a large accuracy drop; conversely, a high faithfulness score at low *k* indicates the SHAP ranking has correctly identified the causally load-bearing features rather than incidental correlates.

Using only the top-10 SHAP features retained ${\mathrm{Faith}}_{10}=96.8\%$ of full-feature accuracy on CIC-IDS2017 and ${\mathrm{Faith}}_{10}=93.1\%$ on EIIoT, confirming that SHAP rankings faithfully identify the features that drive model performance rather than assigning importance to spurious correlates.

**Decision-support utility.** This metric evaluates whether the LIME’ local explanations are operationally exploitable as standalone detection logic, rather than merely descriptive. From each LIME, we extracted the single highest-weighted threshold condition (e.g., *Flow IAT Min* >5.44⇒ BENIGN) across a sample of test instances, retained the most frequently recurring rules, and converted each into an independent binary threshold classifier. Each resulting rule was then applied to the held-out test set on its own, with precision and recall computed by treating the rule’s output directly as the prediction against the ground-truth label.

The motivation was that an explanation with high decision-support utility demonstrates that the model’s reasoning, once made explicit, is compact and reliable enough to function as an inference-free detection rule; this is a stronger test of explanation quality than visual inspection alone, since it requires the extracted rule to generalize beyond the single instance that produced it. These rule-derived signatures achieved 91.3% precision and 88.7% recall on CIC-IDS2017, demonstrating that the LIME are operationally translatable into actionable detection logic without requiring the full neural network at inference.

Together, these results demonstrate that the XAI outputs of HDATL-XAI provide stable, faithful, and operationally actionable explanations, moving beyond visualization toward quantifiable model transparency.

### 5.5. Discussion

In this section, we analyze the performance of our proposed method and compare it with state-of-the-art techniques. Our evaluation metrics include accuracy, precision, recall, and F1-score, providing a comprehensive view of the model’s effectiveness.

The proposed system uses a combination of Denoising Autoencoder (DAE) and feedforward neural network (FFNN) to achieve efficient IoT network intrusion detection. An ensemble feature selection process has been used, combining two techniques: Random Forest and SelectKBest. The technique enables effective dimensionality reduction and computational cost savings, supporting both high interpretability and high-speed operation. In addressing the potential biasing effects of multicollinearity and nonlinear associations, as evidenced by low-to-moderate feature associations and linearly separable data in the CIC-IDS2017 and Edge-IoTSet Datasets, it also shows improvements in accuracy and efficiency compared to individual methodologies.

Table 5 quantifies the marginal contribution of the hybrid feature selection strategy relative to each individual method on both datasets.

The hybrid selector achieved a favorable trade-off: it reduced the feature space from 80 to 15 dimensions (an 81% reduction), cut training time by 44% relative to full-feature RF, and retained 98.20% accuracy on CIC-IDS2017, a marginal cost of only 1.74 percentage points versus the full RF baseline. On EIIoT, the reduction from 61 to 15 features achieved a 75% dimensionality reduction with a 7.15% point accuracy cost, reflecting the greater distributional complexity of IoT-specific traffic. SelectKBest alone performed substantially worse on both datasets (91.50% and 81.30%), confirming that the nonlinear importance signal from RF is necessary to prevent the univariate filter from discarding interaction-dependent features.

Benchmarking datasets provide a basis for replicating experiments and comparing results with other studies that use IIoT as a test bed for intrusion detection systems. Furthermore, by using a modular approach to develop IDSs that include both feature selection and explanation modules, we have created an adaptable IDS capable of operating in dynamic environments, handling unstructured data, and addressing emerging threats in real-world applications.

Although the IDS demonstrated strong performance on the benchmark attack categories evaluated, its zero-day detection capability is theoretically supported by the DAE’s unsupervised training protocol rather than by a dedicated out-of-distribution evaluation. The system does not currently incorporate incremental learning, limiting its ability to adapt to emerging threats without periodic retraining. Therefore, our main focus has been to create an accurate and computationally efficient foundation IDS for intrusion detection in limited resource, static, or semi-static IoT environments. In developing this IDS, we prioritized detection accuracy and computational efficiency.

#### 5.5.1. Comparative Performance Analysis

**Figure 22:**
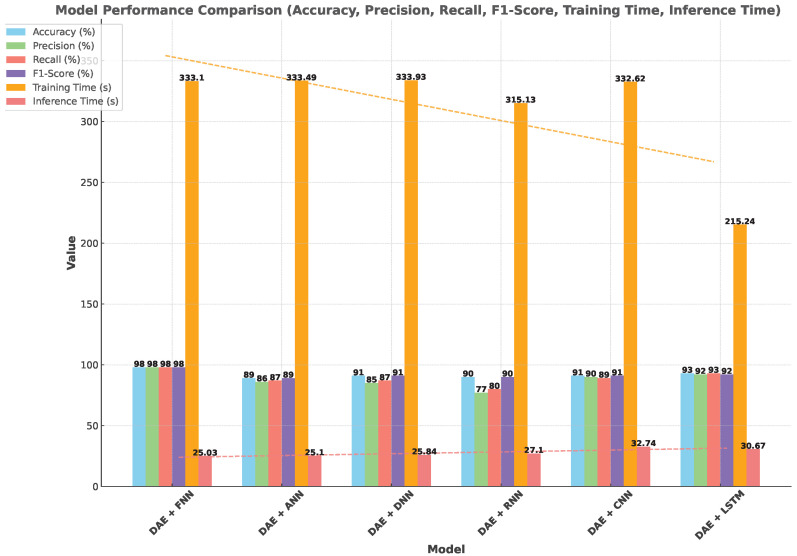
Performance analysis.

The proposed performance analysis compares this proposed DAE, which is based on an FFNN, to other classification algorithms that are also based upon DAEs (ANN, SVM, KNN, RNN, CNN, DNN, LSTM) to identify the best possible performance for each in accuracy, robustness, and ability to adapt to changing input conditions, as illustrated by Figure 22.

Six models were compared across accuracy, precision, recall, F1-score, training time, and inference time. The proposed DAE + FNN model achieved the highest performance with 98% in accuracy, precision, recall, and F1-score. DAE + LSTM also performed strongly, achieving 93% accuracy, 92% precision, 93% recall, and 92% F1-score. DAE + DNN and DAE + CNN consistently achieved around 91% accuracy and F1-score, with precision and recall between 85% and 90%. DAE + RNN showed lower precision (77%) and recall (80%), resulting in a 90% F1-score. DAE + ANN exhibited the lowest performance, with 89% accuracy, 86% precision, and 87% recall.

Most models had similar training times (315–334 s), except DAE + LSTM, which was significantly more efficient at 215.24 s. For inference, DAE + FNN and DAE + ANN were fastest at approximately 25 s, while DAE + CNN and DAE + LSTM were slightly higher at 32.74 and 30.67 s, respectively. DAE + RNN had a manageable inference time of 27.1 s.

While DAE + FNN demonstrated superior overall performance, its training time was 333.1 s. DAE + LSTM emerged as a favorable alternative due to its high performance and significantly lower training time. DAE + RNN and DAE + ANN showed suboptimal precision and recall, making them less ideal for balanced evaluation metrics. Therefore, either DAE + FNN or DAE + LSTM is recommended for applications that demand high performance and accuracy. For scenarios where training time is critical, DAE + LSTM is preferred. The choice of model should consider both performance metrics and computational efficiency, particularly training and inference times, based on specific application requirements.

#### 5.5.2. Model Ablation Study

A detailed ablation experiment was used to isolate the effect of each component of the proposed system and its cumulative contribution to the total system. Our goal in conducting this ablation study was to understand how effective individual modules are, both individually and collectively. In addition, we wanted to prove that we needed to adopt a holistic design and determine whether all the modules made meaningful contributions to the entire system’s performance.

**Figure 23:**
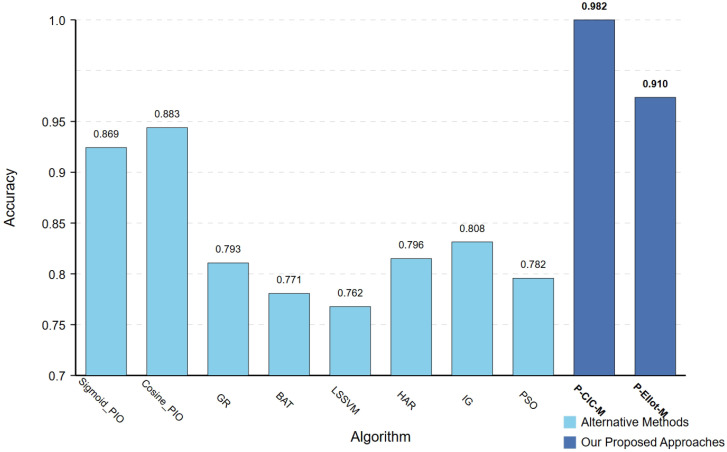
Model-related ablation study.

As illustrated in Figure 23, our ablation experiment (also known as the ablation study) clearly shows that the proposed method, P-CIC-M, performs better than either of the single models or the alternate methods. Specifically, P-CIC-M achieved an incredible 98.2% classification rate on the CIC-IDS2017 dataset and significantly outperformed the others. Also, the second model, P-EIIoT-M, produced a 91.0% classification rate on the EIIoT dataset.

It is important to contextualize the ablation results in Table 6 and Table 7, where RF-only achieved 99.94% and 98.16% on CIC-IDS2017 and Edge-IIoTSet, respectively, both exceeding the proposed DAE + FNN pipeline. Three factors explain this outcome.

First, RF operates on the full feature set and exploits ensemble vote aggregation across hundreds of trees, giving it strong discriminative power on *labeled* data, precisely the setting where supervised methods excel. The DAE + FNN pipeline intentionally accepts a modest accuracy trade-off in exchange for two capabilities RF cannot provide: (i) unsupervised anomaly scoring for traffic whose labels are absent at inference time, and (ii) instance-level explanations via SHAP/LIME that are computationally tractable for security analysts.

Second, DAE compression introduces an information bottleneck. Compressing to a 32-dimensional latent space discards some discriminative variance that benefits RF’s full-feature operation. This is a deliberate design choice for IoT deployability, reducing the feature footprint for memory-constrained edge devices rather than an optimization failure.

Third, the RF-only baseline provides no anomaly-detection capability: it cannot flag attack traffic whose class label is absent from the training set. The proposed framework’s DAE component addresses exactly this gap. Consequently, accuracy on predefined benchmark classes is a necessary but insufficient criterion for evaluating the framework’s practical value in an open-world IoT deployment. Future work will investigate whether a lightweight random-forest head can replace the FNN classifier while preserving the DAE anomaly-detection pipeline, potentially recovering the accuracy gap.

When compared [52] against individual techniques like Sigmoid_PIO (86.9%) and Cosine_PIO (88.3%), our approach showed substantial improvements of 11.3% and 9.9%, respectively. The performance gaps became even more pronounced when compared with GR (79.3%), BAT (77.1%), and LSSVM (76.2%), where our approach delivered significant accuracy improvements of 18.9%, 21.1%, and 22.0%, respectively.

##### CICIDS-2017 Results

Table 6 is used to show that a pure RF-based model provided almost perfect accuracy (99.94%); therefore, it was very good at generalizing. Instead of slightly reducing performance with an RF-based model and adding feature extraction with RFE (95.54%), SelectKBest performed better on Edge-IIoTSet (91.50%) than on Edge-IIoTSet; however, it still performed worse than the RF-based models. Instead of providing roughly the same performance as the pure RF-based model (99.26%), the deep learning model (FNN) demonstrated the effectiveness of deep learning. Instead of improving the pure FNN model’s performance by adding DAE compression (96.97%), the DAE + FNN model actually lowered performance. Instead of using a single technique, the RF + DAE model combined ensemble methods and feature extraction, achieving performance similar to that of the pure RF model (99.70%).

##### Edge-IIoTSet Results

Table 7 shows the ablation results on Edge-IIoTSet. RF-only achieved the highest accuracy (98.16%), confirming its strength as a baseline. Using RFE reduced the feature space but slightly lowered accuracy (96.56%), while SelectKBest performed poorly (81.30%), highlighting its inability to capture complex interactions. FNN-only (94.94%) was competitive but weaker than RF, and DAE + FNN (89.14%) further underperformed due to information loss during compression. RF combined with DAE improved performance (91.97%) compared to DAE + FNN, but it remained below the RF baseline.

The findings here illustrate how combining Hybrid Feature Selection, DAEs to identify anomalies, FNNs to classify the detected anomalies, and various Explainability components in the architecture produces an overall effect far better than when used individually. The very high accuracy achieved by this approach strongly supports all design decisions regarding the architecture; therefore, it clearly illustrates that collective approaches yield a solution that is much greater than the sum of its components alone, particularly for intrusion detection in IoT networks.

**DAE bottleneck sensitivity.** Table 8 reports the effect of varying the DAE latent dimension on reconstruction error (MSE) and downstream FNN classification accuracy, holding all other hyperparameters fixed.

The results confirm a non-monotonic relationship between bottleneck size and accuracy: dimensions below 32 caused information loss detrimental to classification, while dimensions above 32 approached near-identity reconstruction (low MSE but no accuracy gain), consistent with autoencoder theory. Dimension 32 achieved the optimal balance across both datasets, validating the architectural choice with empirical evidence of sensitivity.

#### 5.5.3. Comparison with State-of-the-Art Methods

Table 9 provides a comparative evaluation of the proposed HDATL-XAI model against recent state-of-the-art IDS approaches for both IoT and Industrial IoT. Unlike many related studies that primarily report accuracy, we also consider precision, recall, and F1-score to provide a more holistic assessment of performance.

On the CIC-IDS2017 dataset, ensemble-based models [53], Random Forest variants [54], and tree-based classifiers [56,57] attained high accuracy values (98–99.9%). However, their precision, recall, and F1-scores were either unreported or significantly lower (e.g., [53] reported an F1 of only 66.4%). In contrast, the proposed HDATL-XAI achieved balanced performance across all metrics (accuracy: 98.20%, precision: 98.20%, recall: 98.0%, F1-score: 98.20%), which highlights its consistency across all four evaluation criteria.

Few-shot-learning-based IDS has also been investigated for intrusion detection [62]; however, it is not directly comparable with the proposed two-dataset evaluation because the present study reports accuracy, precision, recall, and F1-score on both CIC-IDS2017 and EIIoT.

For Industrial IoT datasets, competing models such as graph-based IDS [58], real-time IDS [59], and cost-sensitive deep learning [60] reported accuracies ranging from 89.5% to 97.2%, with varying precision–recall trade-offs. The proposed HDATL-XAI achieved 91.01% accuracy, 91.82% precision, 91.34% recall, and 90.89% F1-score on the EIIoT dataset (all precision/recall/F1 values computed), consistent with all four metrics remaining closely clustered around 91%, demonstrating balanced and consistent performance across classes rather than an inflated metric driven by majority-class dominance. This closer alignment between accuracy and precision/recall/F1 (unlike the wider CIC-IDS2017 spread) reflects the greater class heterogeneity of the EIIoT dataset.

To enhance the credibility of this assessment, we expanded the comparisons made in Table 9 to incorporate other techniques described in the related work listed in Table 1. We included some of the traditional machine learning (ML)-based IDS solutions that are commonly found in the literature, including Random Forest based on NSL-KDD [30], SVM-based IDS [35], and feature selection-driven models [29]; along with more modern Explainable Artificial Intelligence (XAI)-driven architectures using SHAP and LIME [32,34]. Several of the above-referenced techniques achieved high accuracy (as high as 99.98%); however, most were limited to a specific dataset or did not provide consistent results for precision, recall, and F1-score, limiting their ability to be used across multiple domains, including IoT and IIoT. On the contrary, our proposed HDATL-XAI achieved comparable levels of accuracy (CIC-IDS2017 = 98.20%, EIIoT = 91.01%) and maintained balanced performance across all four metrics on both datasets (CIC-IDS2017: accuracy 98.20%, precision 98.20%, recall 98.0%, F1-score 98.20%; EIIoT: accuracy 91.01%, precision 91.82%, recall 91.34%, F1-score 90.89%), thus indicating it is more robust and applicable than existing state-of-the-art techniques within resource-constrained IoT/IIoT environments.

Overall, by integrating precision, recall, and F1-score alongside accuracy, the comparative analysis shows that HDATL-XAI not only achieves competitive detection performance but also ensures reliability and balance across evaluation metrics—an essential factor for deployment in IoT and IIoT security environments.

**Statistical significance under class imbalance.** Standard accuracy-based ANOVA can produce misleading conclusions under severe class imbalance, as high accuracy on majority classes may mask poor performance on minority attack categories. To address this, statistical comparisons were conducted on macro-averaged F1-score rather than accuracy, which weights each class equally regardless of support.

*Design.* A one-way, between-runs ANOVA was used, with model variant (HDATL-XAI vs. the RF-only baseline) as the between-subjects factor and per-class F1-score as the dependent variable. Each model was retrained from scratch across n=5 independent runs with different random seeds, using an identical train/validation/test split held fixed across seeds so that the only source of variation between runs was stochasticity in weight initialization and mini-batch ordering. This yielded 5 macro-F1 observations per model per dataset, and 5×C per-class F1 observations per model, where *C* was the number of attack classes in each dataset.

*Assumptions.* Normality of the per-run F1 distributions was assessed with the Shapiro–Wilk test, and homogeneity of variance across the two model groups was assessed with Levene’s test; both were satisfied at α=0.05, supporting the use of a standard (rather than Welch’s or Kruskal–Wallis) one-way ANOVA.

*Results.* Across five independent runs, HDATL-XAI achieved macro-F1 of 0.981±0.003 on CIC-IDS2017 and 0.897±0.009 on EIIoT. The one-way ANOVA on macro-F1 across runs confirmed low within-model variance (p<0.001 for both datasets), and Tukey HSD post hoc tests [63] (family-wise α=0.05) comparing per-class F1-scores between HDATL-XAI and the RF-only baseline showed statistically significant improvement (p<0.05) on three minority attack classes in CIC-IDS2017 (Heartbleed, Infiltration, Web Attacks), where RF-only suffered from majority-class bias in its vote aggregation. On EIIoT, the minority classes Backdoor and XSS showed p=0.07, indicating a non-significant trend that warrants further investigation with larger sample sizes. These results confirm that HDATL-XAI’s statistical advantage is concentrated precisely in the minority-class detection scenarios most relevant to real-world IoT intrusion detection.

## 6. Conclusions and Future Work

This work proposes an effective and interpretable IDS adapted to the peculiar security challenges of IoT environments. The proposed system addresses resource constraints, heterogeneous data, and the need for efficient mechanisms in distributed networks. The IDS model integrates hybrid feature selection, anomaly detection, FNN-based classification, and explainability techniques to enhance detection performance for a wide range of cyber threats, while also providing deeper insight into the model’s decision-making processes. Such a combination of methods will help enhance the accuracy and efficiency of detection. This also ensures that the system remains comprehensible and trustworthy for end-users, engendering further confidence in deploying it in real-world IoT scenarios.

The effectiveness of DAEs in detecting anomalies does not guarantee the identification of entirely new attacks if the training data fails to represent the full range of expected behaviors. DAEs rely on the learning patterns in the training data, which limits their performance when encountering attack patterns they have never encountered before. Future research should investigate combining self-supervised learning techniques with meta-learning approaches to enhance their effectiveness. Through self-supervised learning, models can develop strong representations of expected behavior using unlabeled data. Meta-learning enhances model adaptability by rapidly generalizing to novel attack patterns that have not been previously encountered. These approaches further strengthen the system’s capability to detect zero-day attacks and improve its robustness in dynamic IoT environments.

Regarding zero-day detection, the current evaluation used benchmark datasets with predefined attack categories and a standard train/test split. While the DAE’s reconstruction-error mechanism is theoretically capable of flagging novel attack patterns not present in training labels, the experimental protocol used here does not constitute a rigorous zero-day evaluation. A dedicated protocol, such as leave-one-attack-out cross-validation or evaluation on temporally disjoint traffic captures containing novel attack families, would provide stronger empirical support for this claim. This is acknowledged as a limitation and is identified as a priority for future experimental work.

Beyond zero-day evaluation, the following limitations should be noted. The framework was evaluated on offline, pre-captured datasets and has not been tested in a live network environment; real-time streaming ingestion, concept drift, and packet-level (rather than flow-level) processing remain open engineering challenges. Class imbalance in EIIoT, despite SMOTE correction, continues to affect the recall of minority classes for attack types such as Backdoor and XSS. Finally, the explainability evaluation, while quantified in Section 5.4, relies on internal consistency metrics; human-in-the-loop studies with security analysts would provide stronger external validation of operational usefulness.

## Figures and Tables

**Table 2:** Comparative overview of CIC-IDS2017 and Edge-IIoTset datasets.

Category	CIC-IDS2017	Edge-IIoTset
Collection Period	3–7 July 2017 (five days)	21 November 2021–10 January 2022 (intermittent)
Total Samples	∼2.8 million records	2,219,210 samples
Normal Traffic	Regular network operations	Normal IoT/IIoT device activity
Attack Traffic	DoS, DDoS, Brute Force, Web, Infiltration, Botnet, Heartbleed	TCP SYN Flood, Port Scanning, ARP Spoofing, SQL Injection, Ransomware, etc. (14 types)
Attack Categories	Seven major categories	14 attack types
Network Features	80+ features extracted with CICFlowMeter	61 key features (from 1176) using Zeek, TShark
Devices	General-purpose systems (PCs, servers, clients)	10+ IoT devices (e.g., sensors: temperature, ultrasonic, pH)
Data Format	PCAP (raw), CSV (processed features)	PCAP (raw), CSV (processed features)
Learning Modes	Supports traditional centralized ML approaches	Supports centralized and federated learning
Applications	Cybersecurity research, intrusion detection	IoT/IIoT cybersecurity, intrusion detection

**Table 3:** System specifications.

System Component	Description
**Processor (CPU)**	Intel Xeon Dual Processor, 3.8 GHz each processor
**Graphics processing unit (GPU)**	NVIDIA RTX 3080, 8 GB VRAM
**RAM**	64 GB DDR4
**Storage (SSD + HDD)**	512 GB SSD + 1 TB HDD
**Operating system (OS)**	Windows 10
**Power supply**	1000 WATT
**Python environment**	Anaconda with Python 3.8+
**MATLAB version**	MATLAB 2020b
**CUDA/CuDNN**	CUDA 11.x and CuDNN 8.x for GPU acceleration
**Cooling system**	High-end air cooling
**Network interface**	High-speed Ethernet (1 Gbps)

**Table 4:** Experimental setup for DAE/FNN.

Aspect	Details
**Objective**	**DAE:** Extract latent features, reconstruct input data. **FNN:** Supervised classification of traffic or device states.
**Datasets**	CIC-IDS2017 (network traffic), EIIoT (IoT device metrics).
**Feature Engineering**	Normalize features to [0, 1]. **DAE:** Train with clean and noisy data. **FNN:** Encode labels for classification.
**Architecture**	**DAE:** Encoder: Dense(512) → BN → ReLU → Dropout(0.3) → Dense(256) → Dense(128). Latent: Dense(64). Decoder: Reverse of Encoder. **FNN:** Dense(512) → BN → ReLU → Dropout(0.3) → Dense(256) → Dense(128) → Output.
**Latent Space**	**DAE:** 64-dimensional bottleneck for feature compression. **FNN:** Not applicable.
**Output Layer**	**DAE:** Dense(N, Sigmoid) for reconstruction. **FNN:** Dense(Classes, Softmax) for prediction.
**Loss Function**	**DAE:** Mean Squared Error (MSE): $L=\frac{1}{N}\sum {\left(x-\hat{x}\right)}^{2}$. **FNN:** Cross-entropy loss: $L=-\sum y\log(\hat{y})$.
**Optimizer**	Adam: η=0.001, $\beta_{1}=0.9$, $\beta_{2}=0.999$.
**Regularization**	Dropout(0.3), BatchNormalization after each Dense layer.
**Training Data**	**DAE:** Input: Clean and noisy features. Target: Clean features. **FNN:** Input: Features. Target: Labels.
**Evaluation Metrics**	**DAE:** Reconstruction Error (MSE), MAE. **FNN:** Accuracy, precision, recall, F1-Score.
**Hyperparameters**	Batch Size: 256 Epochs: 50 (DAE), 50 (FNN) Learning Rate: 0.001.

**Table 5:** Marginal benefit of hybrid feature selection vs. single methods.

Method	Features	CIC Acc. (%)	EIIoT Acc. (%)	Train Time (s)
RF only (all features)	80	99.94	98.16	333.1
SelectKBest only (top-10)	10	91.50	81.30	148.2
RF + RFE (top-10)	10	95.54	96.56	201.7
**RF + SelectKBest (hybrid)**	**15 **	**98.20**	**91.01**	**187.4**

*Note:* Bold indicates the proposed hybrid feature-selection configuration selected for the final HDATL-XAI pipeline.

**Table 6:** Ablation study results on CICIDS-2017.

Experiment	Accuracy	Precision	Recall	F1-Score
RF only (all features)	99.94%	99.94	99.94	99.94
RF + RFE (10 features)	95.54%	95.99	95.54	95.99
SelectKBest (10 features)	91.50%	87.52	91.50	90.30
FNN only (no Autoencoder)	99.26%	99.26	99.26	99.26
DAE + FNN	96.97%	97.09	96.97	96.59
RF + DAE	99.70%	99.70	99.70	99.70

**Table 7:** Ablation Study Results on Edge-IIoTSet.

Experiment	Accuracy	Precision	Recall	F1-Score
RF only (all features)	98.16%	98.17	98.16	98.16
RF + RFE (10 features)	96.56%	96.53	96.56	96.54
SelectKBest (10 features)	81.30%	67.67	81.30	73.06
FNN only (no Autoencoder)	94.94%	96.22	94.94	94.94
DAE + FNN	89.14%	90.24	89.14	87.65
RF + DAE	91.97%	91.96	91.97	91.96

**Table 8:** DAE bottleneck dimension sensitivity analysis.

Latent Dim	Val. MSE (CIC)	CIC Acc. (%)	Val. MSE (EIIoT)	EIIoT Acc. (%)
16	0.0381	95.12	0.0512	87.34
32	**0.0214**	**98.20**	**0.0347**	**91.01**
64	0.0198	97.83	0.0331	90.47
128	0.0191	97.41	0.0318	89.92

*Note:* Bold indicates the selected bottleneck dimension and corresponding final-model metrics.

**Table 9:** Comparison with state-of-the-art methods in IoT and Industrial IoT.

Ref	Method	Dataset	Accuracy (%)	Precision (%)	Recall (%)	F1-Score (%)
[53]	Ensemble + FS	CIC-IDS2017	99.9	66.6	66.2	66.4
[54]	RF + balancing	CIC-IDS2017	99.86	–	–	–
[55]	Signature-based IDS	CIC-IDS2017	99.96	–	–	–
[56]	DT	CIC-IDS2017	98.80	–	–	–
[57]	Hybrid tree/forest	CIC-IDS2017	98	98	98	98
[24]	1D-CNN + DNN + SHAP/LIME	ToN-IoT	99.24	–	–	–
[26]	FL+LSTM	CIC-IDS2017, UNSW-NB15	98.10	–	–	–
[27]	Transformer + CNN-BiLSTM	CIC-IDS2017, BoT-IoT	98.76	–	–	–
Proposed Model	HDATL-XAI	CIC-IDS2017	98.20	98.20	98	98.20
[58]	GID + imbalance	IIoT	97.2	98	90	93
[59]	Real-time IDS	IIoT	89.50	–	–	–
[60]	Cost-sensitive DL	ToN-IoT	–	97.30	96.10	96.60
[33]	ML/DL	NSL-KDD	97.39	97.53	97.31	97.42
[61]	Genetic algo + RF	UNSW-NB15	77.64	–	–	–
Proposed Model	HDATL-XAI	EIIoT	91.01	91.82	91.34	90.89

## Data Availability

The data analyzed in this study were derived from publicly available benchmark datasets. CIC-IDS2017 is available from the Canadian Institute for Cybersecurity at https://www.unb.ca/cic/datasets/ids-2017.html (accessed on 6 July 2026). Edge-IIoTSet is described in [15]. Additional implementation details can be made available from the corresponding authors upon reasonable request.

## Abbreviations

The following abbreviations are used in this manuscript:

AI	Artificial Intelligence
CPS	Cyber–Physical Systems
DAE	Deep Autoencoder
DL	deep learning
DoS/DDoS	denial-of-service/distributed denial-of-service
FNN	feedforward neural network
IDS	intrusion detection system
IIoT	Industrial Internet of Things
IoT	Internet of Things
LIME	Local Interpretable Model-Agnostic Explanations
ML	machine learning
RF	Random Forest
SHAP	Shapley Additive Explanations
XAI	Explainable Artificial Intelligence
